# Molecular cloning and expression analysis of sucrose phosphate synthase genes in cassava (*Manihot esculenta* Crantz)

**DOI:** 10.1038/s41598-020-77669-9

**Published:** 2020-11-26

**Authors:** Tangwei Huang, Xinglu Luo, Maogui Wei, Zhongying Shan, Yanmei Zhu, Yanni Yang, Zhupeng Fan

**Affiliations:** 1 College of Agriculture, Guangxi University, Nanning, 530004 China; 2 State Key Laboratory for Conservation and Utilization of Subtropical Agro-bioresources, Nanning, 530004 China

**Keywords:** Molecular biology, Plant sciences

## Abstract

Sucrose phosphate synthase (*SPS*), a key rate-limiting enzyme in the sucrose biosynthesis pathway in plants, is encoded by a multi-gene family. Until recently, the identification and characterization of the *SPS* gene family have been performed for dozens of plant species; however, few studies have involved a comprehensive analysis of the *SPS* family members in tropical crops, such as cassava (*Manihot esculenta* Crantz). In the current study, five *SPS* genes (*MeSPS1*, *MeSPS2, MeSPS3*, *MeSPS4,* and *MeSPS5*) were isolated from cassava, and their sequence characteristics were comprehensively characterized. These *MeSPS* genes were found distributed on five chromosomes (Chr2, Chr14, Chr15, Chr16, and Chr18). Phylogenetic analysis showed that the *MeSPS* protein sequences were clustered into three families, together with other *SPS* sequences from both dicot and monocot species (families A, B, and C). The spatio-temporal expression pattern analysis of *MeSPS* genes showed a tissue-specific and partially overlapping expression pattern, with the genes mainly expressed in source tissues during cassava growth and development. Correlation analysis revealed that the expression of *MeSPS* genes correlated positively with root starch content, indicating that the expression of *MeSPS* genes might accelerate the rate of starch accumulation in the roots of cassava plants.

## Introduction

Sucrose phosphate synthase (*SPS*; EC2.4.1.14) is a key rate-limiting enzyme in the sucrose biosynthesis pathway in plants^1–5^. *SPS* catalyses the reaction of uridine diphosphate glucose (UDPG) and fructose-6-phosphate to form sucrose-6-phosphate, which is subsequently dephosphorylated and hydrolyzed by sucrose-phosphate phosphatase (*SPP*; EC3.1.3.24), threreby releasing sucrose^6–8^. Previous studies demonstrated that *SPS* activity is significantly correlated with plant growth and development^9–11^, yield and quality of products^12–15^, and plant tolerance to abiotic stresses^16,17^. Further, the *SPS* protein was demonstrated to be encoded by a multi-gene family, with each family containing at least one distinct *SPS* isoform^1,18^. *SPS* genes present in dicotyledonous species are classified into three major families designated as families A, B, and C according to their phylogenetic evolution. In addition, two other subfamilies designated as subfamilies D_**III**_ and D_**IV**_) have been found in monocots^19–21^. The first *SPS* gene to cloned was obtained from maize^22^. To date, numerous distinct *SPS* isoforms have been identified and characterized for dozens of plant species, including *Spinacia oleracea*^23^, *Saccharum officinarum*^24^, *Cucumis melo*^25^, *Ananas comosus*^26^, *Cerasus humilis*^27^, and *Litchi chinensis*^28^ among others. Subsequently, the characterization and expression patterns of *SPS* gene sequences from these plant species have been explored^29^.

Cassava (*Manihot esculenta* Crantz) is a typical perennial crop of the *Euphorbiaceae* family. Owing to its starchy storage roots, cassava is one of the most important and widely cultivated staple food crops for feeding more than 800 million people in tropical and subtropical regions of Africa, Asia, South America, and the Pacific^30^. In cassava storage roots, sucrose is readily hydrolysed into fructose and UDPG, the latter being the immediate precursor of starch synthesis. To date, the physiological function of *SPS* isozyme was comprehensively illustrated in prior studies, but not yet about the molecular bases and expression patterns of *SPS* genes in cassava^31,32^. Thus, it is fundamental paramount for understanding of the *SPS* genes roles in regulating the sucrose biosynthesis and even in the starch accumulation pathway in cassava roots.

In the current study, members of the cassava *SPS* gene family were cloned and characterized from. Our study mainly focused on *SPS* gene cloning, chromosomal localization, exon–intron organization and structural analyses of conserved motifs. Additionally, we investigated the phylogenetic evolution and expression profile of these genes (in different tissues and at developmental stages). Further, the relationship between *SPS* gene expression and starch accumulation in the root was studied by the correlation analysis. Our results provide a solid foundation for a thorough understanding of the physiological role of *SPS* genes in regulating the starch accumulation in the storage roots of cassava.

## Results

### Cloning and sequence characteristics of MeSPS genes

**Figure 1:**
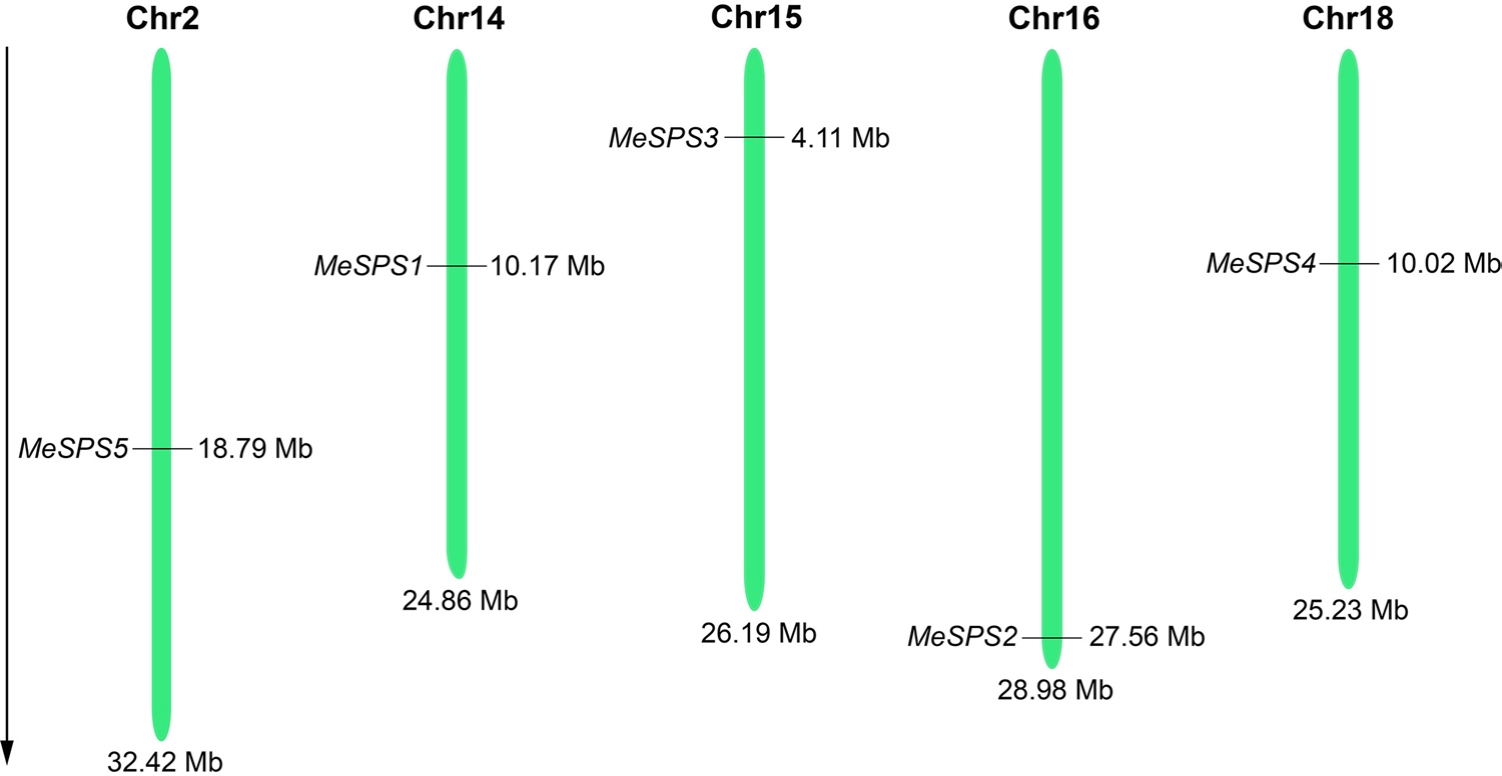
Chromosomal locations of *MeSPS* genes in the cassava genome. Green bars represent the chromosomes model, and the length of five chromosomes are labeled. Black short lines indicate the coordinate of each *MeSPS* gene in the cassava genome. Black arrow on the left indicates direction of gene sequences.

Results of agarose gel electrophoresis showed that the target fragments were effectively amplified via real-time PCR (RT-PCR). The full-length gels are shown in Supplementary Fig. S1. Five SPS genes, hereafter designated as MeSPS1, MeSPS2, MeSPS3, MeSPS4, MeSPS4, and MeSPS5, were isolated from cassava. The characteristics of these five SPS genes are summarized in Table 1. The results showed that the open reading frames (ORF) of the isolated MeSPS genes ranged from 2805 to 3198 bp in the length, while the predicted molecular weights (Mw) for the encoded proteins ranged between 104.12 and 119.46 kDa (934–1065 amino acids, their nucleotide and amino acid sequences are listed in Supplementary Data S1–S2). The theoretical isoelectric point (pI) of the MeSPS proteins ranged from 5.81 to 6.76. Furthermore, in order to ascertain the genomic organization of MeSPS genes, their locations and coordinates on the cassava chromosomes were mapped, and they were found to be distributed on chromosomes Chr14, Chr16, Chr15, Chr18, and Chr2, respectively (Table1; Fig. 1).

**Table 1:** Sequence information and characteristics of *SPS* genes in cassava.

Gene name	GenBank ID	Gene coordinates (5′–3′)	ORF length (bp)	Protein length (a.a)	Predicted Mw (kDa)	Theoretical *pI*
*MeSPS1*	KX822780	10168870–10177066	3174	1057	118.25	6.19
*MeSPS2*	XM021748682	27559266–27563572	2805	934	104.12	5.81
*MeSPS3*	MK181569	4112076–4118991	3198	1065	119.46	6.08
*MeSPS4*	MK181570	10012671–10019724	3060	1019	115.29	6.76
*MeSPS5*	XM021740620	18791429–18799770	3066	1021	115.75	6.26

**Figure 2:**
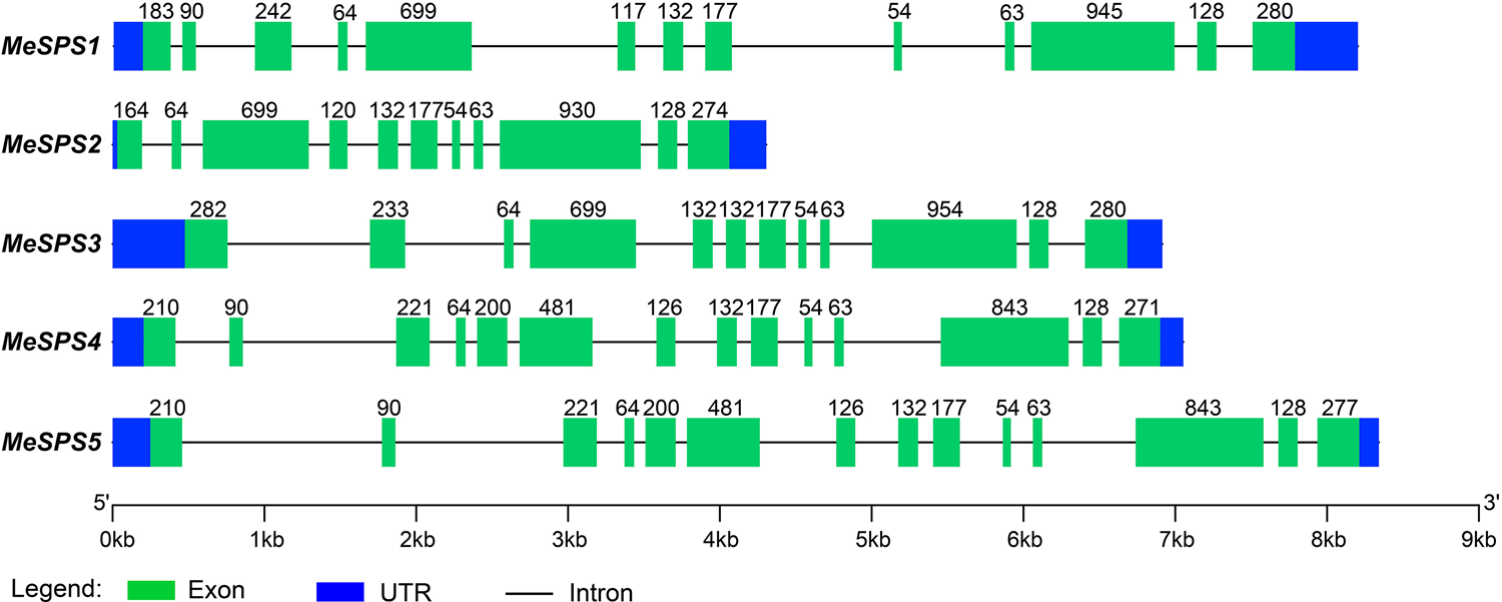
Exon–intron structure of *MeSPS* genes. Green boxes indicate exons. Black lines between green boxes represent introns. Blue boxes represent the untranslated regions (UTRs). Numbers represent the length of exons. Gene length can be estimated using the scale at bottom.

**Figure 3:**
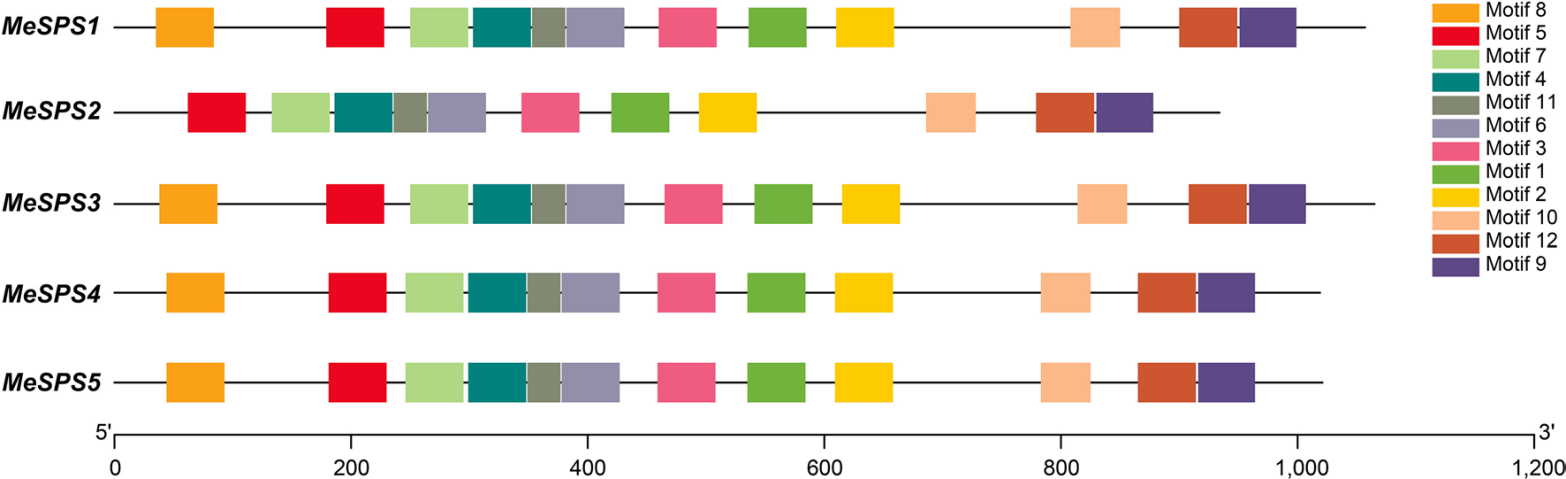
Conserved motif distribution in cassava *MeSPS* proteins. Motifs were analyzed by the MEME program and TBtools v0.6 software (Supplementary Table S1). Conserved motifs 1–12 are marked by different colours. The lengths of proteins and motifs can be estimated using the scale at bottom.

Structural analysis of exons–introns in MeSPSs indicated that MeSPS1 contains thirteen exons and twelve introns, MeSPS2 includes eleven exons and ten introns, MeSPS3 has twelve exons and eleven introns, and MeSPS4 and MeSPS5 are each composed of fourteen exons and thirteen introns. The length of exons differed among MeSPS genes (Fig. 2). To further analyse the diversity of the MeSPS proteins, conserved motifs were predicted using the MEME program (http://meme-suite.org/tools/meme) and Toolbox for Biologists (TBtools version 0.6) software (http://www.tbtools.com/). We distributed 12 putative conserved motifs (referred to as motifs 1–12) in the same direction in all MeSPS protein sequences, furthermore, most of these conserved motifs were located within the C-terminal region (Fig. 3). The average length of these motifs was 50 amino acids, except for motifs 9, 10, and 11, which contained 49, 43, and 29 amino acid residues, respectively (the features of these motifs are listed in Supplementary Table S1).

### Multiple alignment and phylogenetic analyses

**Figure 4:**
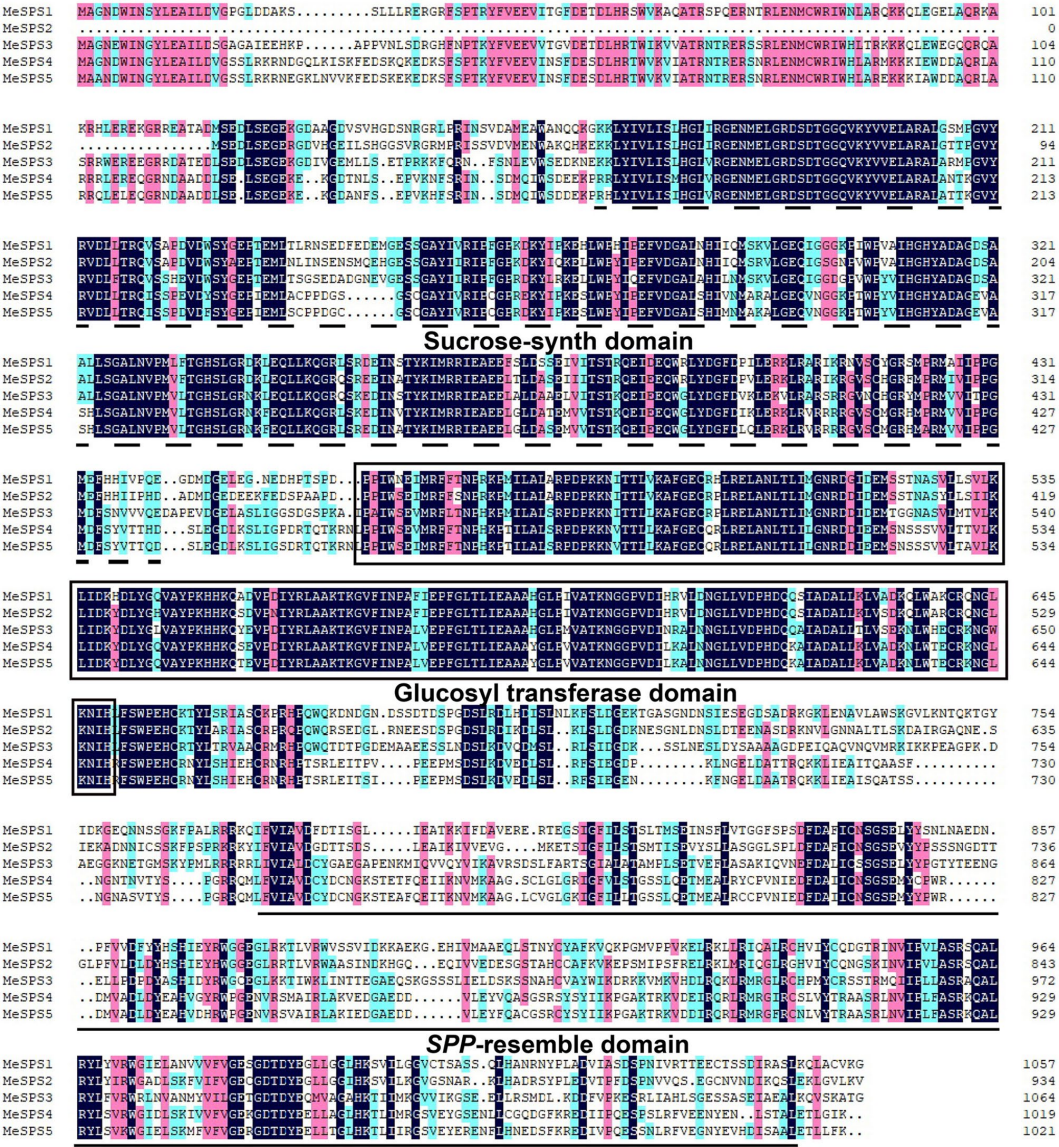
Multiple alignments of MeSPS deduced amino acid sequences. The alignment was performed using the multiple alignment program of DNAMAN v7.0. Consensus residues are in black, residues that are ≥ 75% identical among the aligned sequences are in pink, and residues that are ≥ 50% identical among the aligned sequences are in blue. Three characteristic functional domains were marked out. Black dotted lines indicate Sucrose-synth domain. Solid line boxes indicate Glycosyl transferase domain and black underlines indicate SPP-resemble domain.

Multiple alignment analysis of the identified MeSPS sequences showed that the isolated MeSPS genes share a high sequence identity at both nucleotide (46.45–80.81%) and amino acid (47.90–91.09%) levels (Table 2). Among them, MeSPS4 and MeSPS5 showed the highest homology levels, with 80.81% and 91.09% at nucleotide and amino acid levels, respectively, compared with the other paralogs. Moreover, analysis of conserved domains indicated that three conserved domains—the sucrose synthase, glucosyl-transferase and resemble SPP domains, which have been catalogued as typical plant SPS domains—were also detected in all isolated MeSPS genes, using the SMART (http://smart.embl-heidelberg.de/) and the InterPro (http://www.ebi.ac.uk/interpro/search/sequence/) databases (Fig. 4). These results led to the suggestion that the five isolated MeSPS genes encode different SPS isozymes in cassava.

**Table 2:** Identical coefficients of *MeSPS* amino acid and nucleotide sequences.

	Identity of amino acid sequences (%)				
	*MeSPS1*	*MeSPS2*	*MeSPS3*	*MeSPS4*	*MeSPS5*
**Identity of nucleotide sequences (%)**					
*MeSPS1*	–	65.88	57.67	54.70	54.79
*MeSPS2*	57.93	–	50.14	47.90	47.94
*MeSPS3*	50.19	50.67	–	58.10	56.70
*MeSPS4*	47.75	47.20	60.54	–	91.09
*MeSPS5*	52.21	46.45	54.02	80.81	–

**Figure 5:**
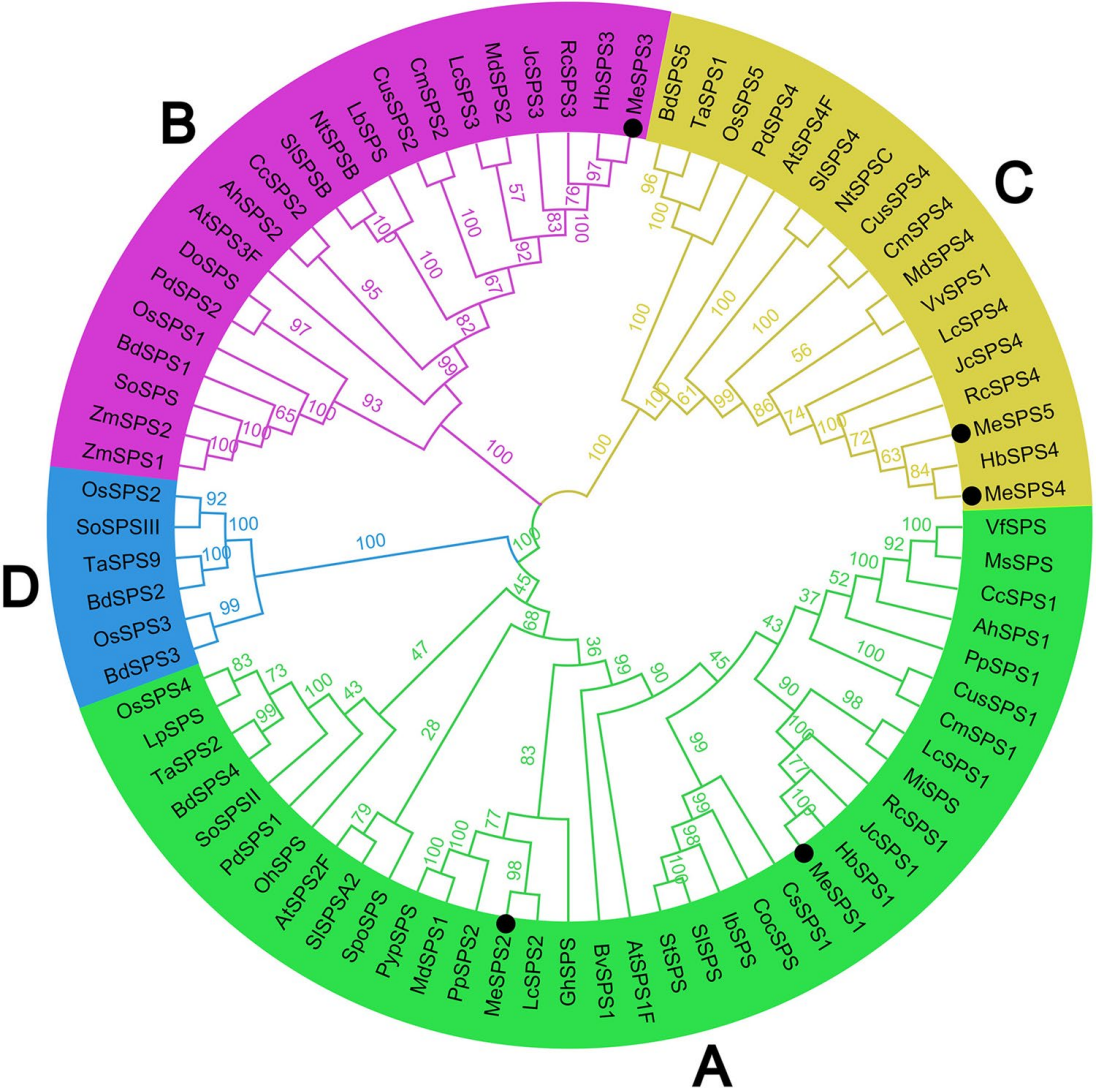
Phylogeny of SPS proteins from cassava and other plant species (abbreviations and sequences of other plant SPS proteins are summarized in Supplementary Table S2). Four SPS gene families are distinguished by different colours. Numbers in the branch nodes are bootstrap values. Black dots represent SPS proteins of cassava.

To further illustrate the evolutionary relationship between MeSPSs and SPSs from other plant species, a total number of 80 full-length amino acid sequences from 36 species (comprising dicots and monocots, amino acid sequences summed in Supplementary Table S2) were selected to construct an unrooted phylogenetic tree using the MEGA (version X.0) software (https://www.megasoftware.net/). As shown in Fig. 5, all SPS proteins were clearly divided into four major families, hereafter designated as A, B, C, and D, with 100% bootstrap values. Families A, B, and C consist of SPS protein sequences from dicots and monocots plant species, whereas family D comprised only monocotyledonous sequences. The distribution of the seven cassava SPS proteins was the following: MeSPS1 and MeSPS2 belong to family A, MeSPS3 was assigned to family B, and MeSPS4 and MeSPS5 were clustered in family C.

### Expression patterns of MeSPS genes in different tissues and at developmental stages

**Figure 6:**
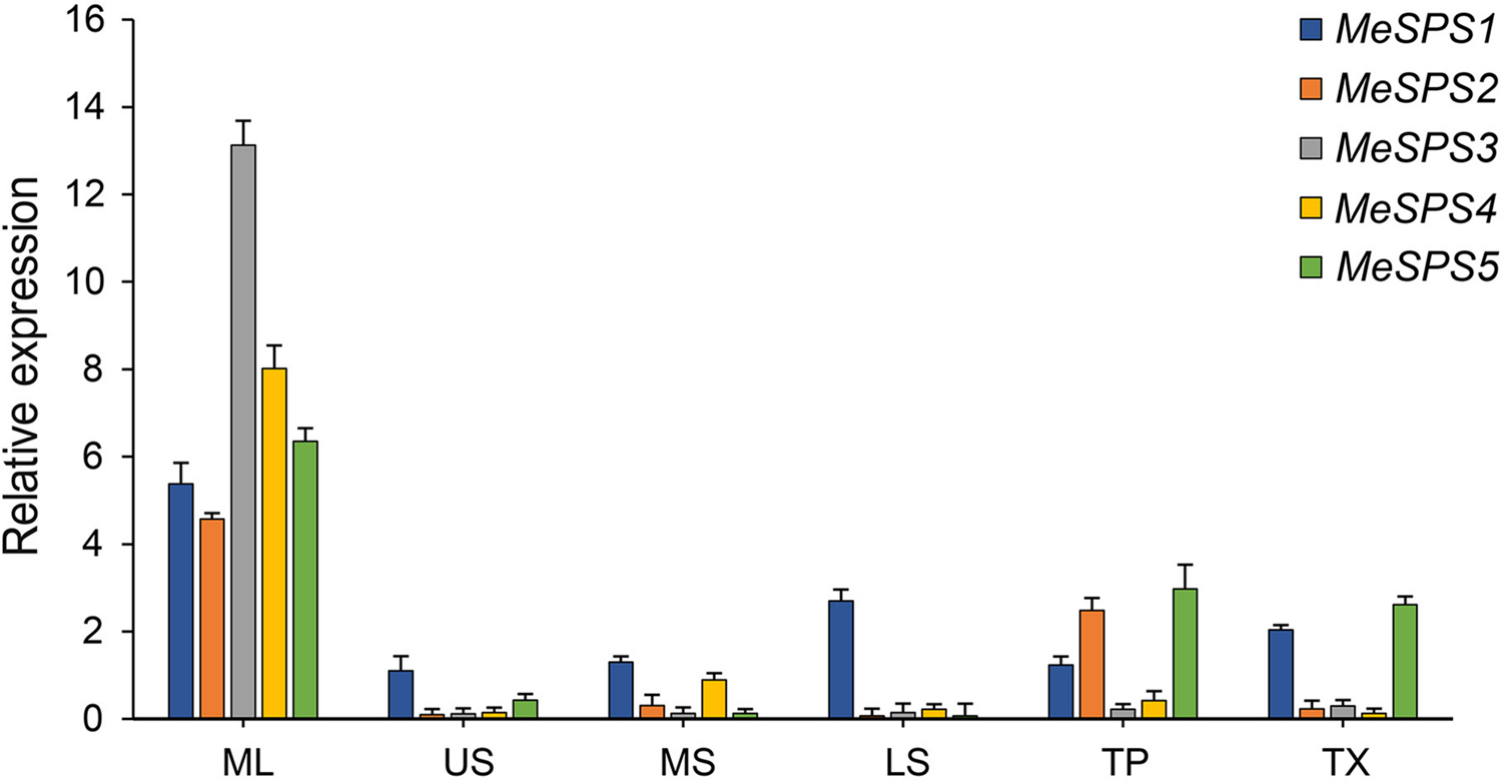
Expression patterns of MeSPS genes in different cassava tissues at root maturation stage. ML, US, MS, LS, TP, and TX represent mature leaves, upper part of stem, middle part of stem, lower part of stem, tuber phloem, and tuber xylem, respectively.

In pursue of the physiological function of each gene in the MeSPS gene family, the spatio-temporal expression pattern of the isolated MeSPS genes were profiled in six cassava tissues—mature leaves (ML), the upper part of stem (US), the middle part of stem (MS), the lower part of stem (LS), tuber phloem (TP), and tuber xylem (TX)—at the root expansion stage (180 DAP, 2017) via real-time fluorescent quantitative PCR (RT-qPCR) analysis. Figure 6 indicated the expression pattern of MeSPS genes in each tissue. Overall, the expression of MeSPS genes revealed a tissue- and organ-specific pattern, where the five target genes showed a relatively low level of expression in US, MS, LS, TP, and TX, but had an abundant expression in the ML. Specifically, MeSPS3 revealed the highest level of expression in ML (i.e., more than 13-fold higher) compared with the other MeSPS genes. These findings suggested that cassava SPS genes were mainly expressed in source tissues rather than in transport or sink tissues.

**Figure 7:**
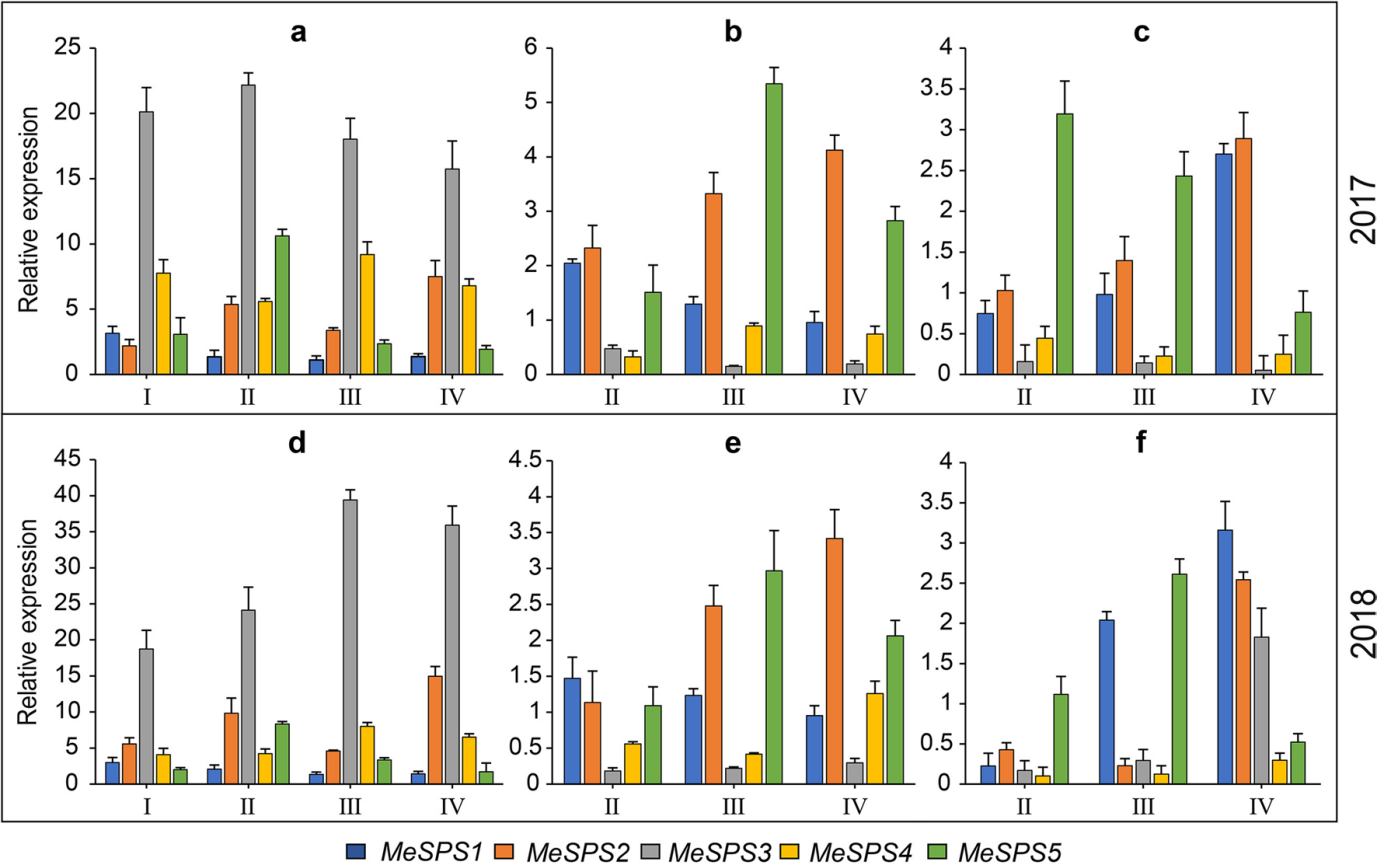
Differential expression of MeSPSs in leaves (**a**,**d**), phloem of tubers (**b**,**e**), and xylem of tubers (**c**,**f**) at different stages. I, II, III, and IV represent the seedling stage, root formation stage, root expansion stage, root maturation stage, respectively.

The periodical variation of MeSPS expression in source (ML) and sink (TP and TX) tissues were also examined via RT-qPCR during four different growth periods: seedling stage (I), root formation stage (II), root expansion stage (III), and root maturation stage (IV) in 2017 and 2018. As shown in Fig. 7, all MeSPSs were relatively highly expressed (MeSPS3 reached near 40-fold) in source tissues (ML). These patterns are consistent with the expression profiles observed in a wide range of tissues (Fig. 6). Overall, MeSPS gene expression showed dynamic changes in both 2 years. In ML, MeSPS1 maintained a low and stab expression pattern during all growth stages in the 2 years. The expression patterns of MeSPS2 and MeSPS5 exhibited a similar trend (e.g., peaking at stage II and then gradually decreasing during the later stages of growth). In turn, MeSPS3 was first up-regulated and peaked at stage II in 2017, and at stage III in 2018. Meanwhile, MeSPS4 first increased to a peak at stage III and then gradually decreased at later stages in both 2 years (Fig. 7a,d). In TP, MeSPS1 showed a continuous down-regulation during the growth stages analysed, whereas MeSPS2 and MeSPS5 revealed a significant up-regulation to a peak at stage IV in both 2 years. In contrast, MeSPS3 was only weakly expressed across growth stages, and MeSPS4 was slightly up-regulated during the experimental period (Fig. 7b,e). Finally, as for TX, MeSPS1 and MeSPS2 showed a rapid increase from the beginning and peaked at stage IV in both 2 years. In turn, MeSPS3 showed a low level of expression in 2017, and a high expression level at stage IV in 2018. MeSPS4 was weakly expressed throughout the experimental period, while MeSPS5 reached a peak at stage II in 2017, and at stage III in 2018, but rapidly decreased at later stages in both years (Fig. 7c,f). Altogether, these results showed that none of the cassava tissues under study expressed only one MeSPS gene during cassava growth and development.

### Physiological determination and correlation analysis

**Figure 8:**
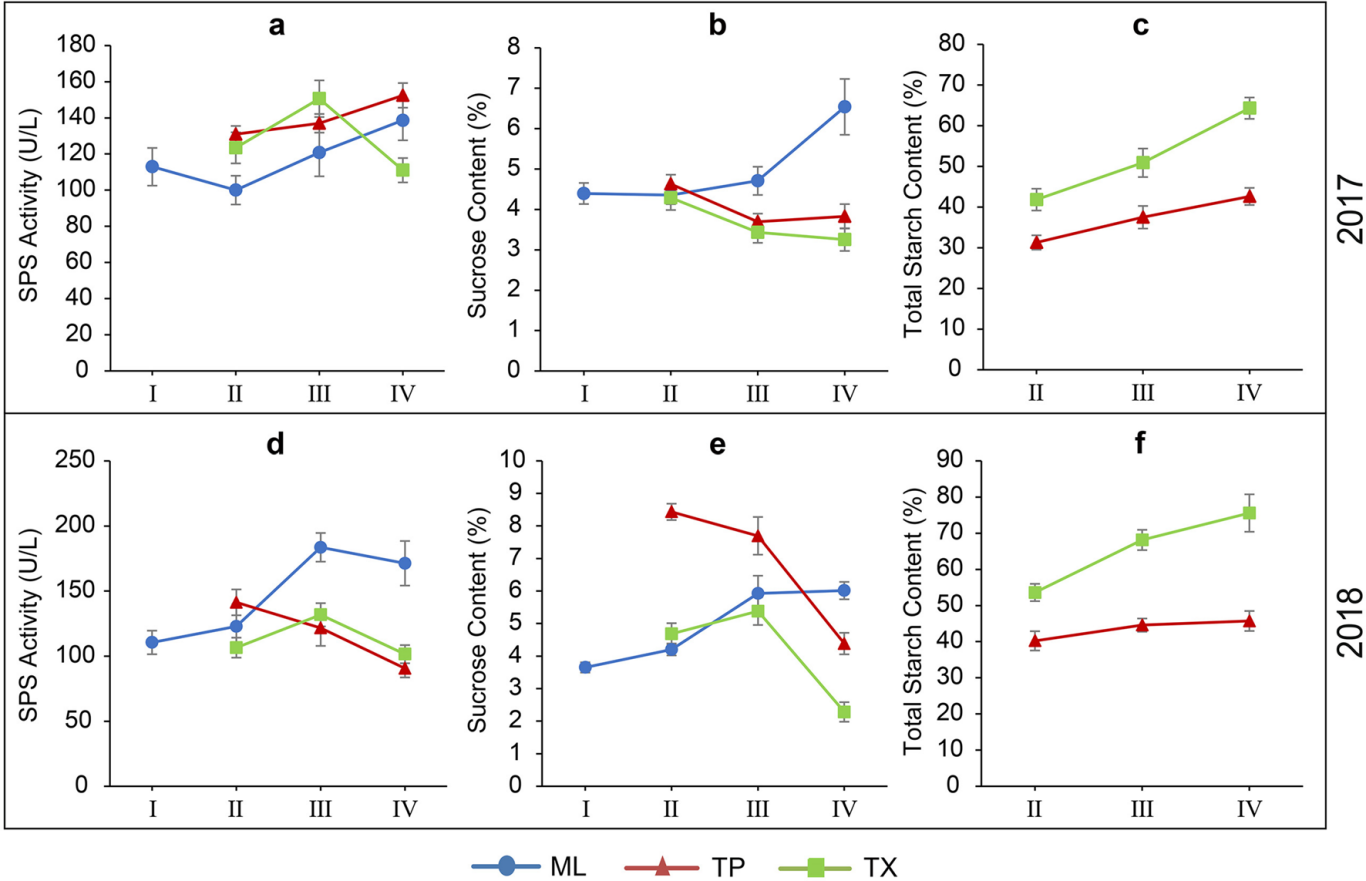
Variation of SPS activity (**a**,**d**), sucrose content (**b**,**e**), and total starch content (**c**,**f**) in different tissues of cassava during growth. I, II, III, and IV represent seedling stage, root formation stage, root expansion stage, root maturation stage, respectively. ML, TP, and TX represent mature leaves, tuber phloem, and tuber xylem, respectively.

*SPS* activity and sucrose content in ML, *SPS* activity, sucrose content, and total starch content in TP and TX, were determined at four growth stages in 2017 and again in 2018. As shown in Fig. 8, *SPS* activity (Fig. 8a,d) and sucrose content (Fig. 8b,e) gradually increased in ML throughout growth in the two experimental years. Conversely, in TP, *SPS* activity in 2018 (Fig. 8d) and sucrose content (Fig. 8b,e) decreased gradually in both years. However, *SPS* activity in 2017 (Fig. 8a) increased gradually, and this trend was similar with that of the total starch content in both years (Fig. 8c,f). On the other hand, in TX, *SPS* activity in the 2 years (Fig. 8a,d) and sucrose content in 2018 (Fig. 8e) exhibited a similar behavior without any particular trend, whereas sucrose content gradually decreased in 2017 (Fig. 8b). While total starch content increased consistently throughout growth both in 2017 and 2018 (Fig. 8c,f).

Correlation analysis was performed to determine the relationships among MeSPS gene expression level, sucrose biosynthesis and starch accumulation in ML, TP, and TX, respectively. Correlation coefficients are summarized in Table 3. Overall, all examined physiological indexes were significantly correlated with the expression level of one or more MeSPS genes in both years. Specifically, the expression level of MeSPS1 in TX was significantly and positively correlated with total root-starch content (both in TP and TX) both in 2017 and 2018. Similarly, the expression level of MeSPS2 and MeSPS5 in TX were positively correlated with total starch content in TX. Finally, the expression level of MeSPS3 in TP, and those of MeSPS3 and MeSPS4 in TX were also positively correlated with total starch content in TP. These findings clearly suggest that starch accumulation in the storage root of cassava might be promoted by the expression of MeSPS genes during development.

**Table 3:** Coefficients of correlation between expression levels of MeSPS genes related to SPS activity, sucrose and total starch contents in leaves and roots of cassava. ML, TP, and TX represent mature leaves, tuber phloem, and tuber xylem, respectively.

Year	Tissue	Gene expression	ML		PT			XT		
			SPS activity	Sucrose content	SPS activity	Sucrose content	Total starch content	SPS activity	Sucrose content	Total starch content
2017	ML	*MeSPS1*	0.411	0.307	− 0.680*	0.904**	0.895**	− 0.178	0.609	− 0.856**
		*MeSPS2*	0.146	0.587	− 0.058	− 0.652	0.190	0.684*	− 0.111	0.033
		*MeSPS3*	0.912**	0.886**	− 0.673*	− 0.167	− 0.195	0.547	0.224	− 0.408
		*MeSPS4*	0.470	0.032	0.333	0.056	0.275	− 0.767*	− 0.179	0.437
		*MeSPS5*	0.654*	− 0.491	− 0.261	− 0.526	− 0.028	0.401	0.110	− 0.187
	PT	*MeSPS1*	0.024	0.698*	0.778*	− 0.643	0.845*	− 0.323	− 0.676	0.946**
		*MeSPS2*	0.285	0.475	0.734	− 0.515	0.632	− 0.114	− 0.134	0.670*
		*MeSPS3*	− 0.622	0.784*	− 0.609	0.807*	0.718*	− 0.403	0.574	− 0.672*
		*MeSPS4*	0.673*	0.701*	0.301	− 0.953*	0.645	0.320	− 0.440	0.570
		*MeSPS5*	0.365	− 0.137	0.284	0.582	0.066	0.868**	0.080	0.042
	XT	*MeSPS1*	0.895**	− 0.094	− 0.605	− 0.649	0.634*	0.730*	0.108	0.790*
		*MeSPS2*	− 0.168	0.558	− 0.726*	− 0.356	− 0.368	− 0.550	− 0.626	0.903**
		*MeSPS3*	0.345	0.649*	− 0.787*	0.414	− 0.599	− 0.113	0.706	0.600
		*MeSPS4*	0.679	0.167	− 0.506	0.833*	0.858**	− 0.351	0.608	0.699
		*MeSPS5*	− 0.054	− 0.363	− 0.298	0.383	− 0.641	0.040	0.144	0.660*
2018	ML	*MeSPS1*	0.457	0.088	− 0.114	0.180	0.328	− 0.732*	0.869**	0.237
		*MeSPS2*	0.158	0.184	0.312	− 0.190	− 0.104	0.514	− 0.148	− 0.215
		*MeSPS3*	0.936**	0.827**	− 0.769*	− 0.603	0.607	0.146	− 0.157	0.494
		*MeSPS4*	0.608*	0.547	− 0.376	− 0.596	0.597	− 0.561	− 0.772*	0.330
		*MeSPS5*	0.687*	− 0.103	− 0.006	− 0.638	0.407	0.329	0.576	0.271
	PT	*MeSPS1*	− 0.686*	− 0.815*	0.186	0.172	0.637	0.126	0.392	− 0.068
		*MeSPS2*	− 0.771*	− 0.728*	0.601	0.714	− 0.721*	0.296	0.703*	0.747*
		*MeSPS3*	0.749*	0.656	0.304	0.673	0.769*	− 0.289	− 0.538	0.557
		*MeSPS4*	0.839*	0.747*	0.111	0.236	0.478	− 0.699*	− 0.896**	0.252
		*MeSPS5*	0.067	0.286	− 0.240	− 0.458	0.695*	0.726*	0.185	0.635
	XT	*MeSPS1*	0.799*	0.793*	− 0.630	− 0.853*	0.925**	0.018	0.469	0.705*
		*MeSPS2*	0.616	0.433	− 0.529	− 0.068	− 0.011	− 0.749*	− 0.684*	0.886*
		*MeSPS3*	0.944*	0.948*	− 0.235	− 0.479	0.696*	0.558	0.832**	0.326
		*MeSPS4*	0.883*	0.880*	− 0.221	− 0.514	0.701*	0.488	0.746*	0.354
		*MeSPS5*	− 0.507	− 0.414	− 0.655	0.186	− 0.733*	0.363	0.130	0.678*

Significant differences are indicated by asterisks (*P < 0.05, **P < 0.01).

## Discussion

*SPS* have been shown to play an important regulatory role in the sucrose biosynthesis pathway in higher plants. Recently, due to a growing number of high-quality whole genome sequences, various *SPS* genes from numerous plant species were cloned and classified. However, the prevalence and functional diversity of *SPS* gene family members are less well defined in tropical crops such as cassava, which is characterized by a high starch content in the roots. To our knowledge, this is the first report of gene structure, protein motifs, and protein multiple alignments of cassava *SPS* genes.

In the current work, five *SPS* isoforms were isolated from cassava*,* which were designated as *MeSPS1*–*5*, respectively. All *MeSPS* proteins harboured the glucosyl–transferase (N-terminal) and resemble the *SPP* (C-terminal) domains (Fig. 4), that allow the catalytic reactions in the sucrose biosynthesis pathway in higher plants^20,33^. The distribution of exons and introns varied among the *MeSPS* genes, which contained 11–14 exons and 10–13 introns (Fig. 2). Similar findings were reported for other crops, such as wheat^8^, maize^20^, and rice^34^, in which the number of exons and introns in the coding regions also ranged from 11 to 14, and from 10 to 13, respectively. These structures determined similar functions among *SPS* genes of dicot and monocot plant species distantly related in evolutionary history^35–37^.

A comprehensive phylogenetic analysis should help us gain novel insights into both the origins and evolutionary relationships among diverse members of gene family identified, as well as into their potential functions^38^. Based on the phylogenetic analysis of the five cassava *MeSPS* and other *SPS* proteins in this study, four major families were defined, among which, families A, B, and C include *SPS* proteins from dicots and monocots, whereas family D only includes monocot sequences (Fig. 5). These findings further corroborated *SPS* classifications previously reported, and lend support to the proposition that higher plant species may have at least three major families of *SPS* genes^19–21^. Interestingly, the phylogenetic tree constructed herein further revealed that most *MeSPS* proteins were more closely related to the proteins of *RcSPS* (*R. communis*), *JcSPS* (*J. curcas*), and *HbSPS* (*H. brasiliensis*), as these four species belong to the same *Euphorbiaceae* family. These findings are consistent with the conclusions by Bredeson et al.^39^.

In the recent decades, the expression of *SPS* genes has been comprehensively documented for a number of plant species, however, few detailed analyses have been conducted in cassava. In this study, the expression patterns of Me*SPS* genes in different cassava tissues and at different developmental stages were examined. The results showed that all *MeSPS* genes were mainly expressed in leaves, compared with other tissues, and there was at least one *MeSPS* gene predominantly expressed in source and sink tissues in both experimental years (Fig. 7), indicating that *MeSPS* genes exhibited a tissue-specific and partially overlapping expression pattern during cassava growth and development. Similar spatio-temporal expression of *SPS* genes in various other plants have been previously expounded. For instance, the expression of *SPS* genes from *Citrus unshiu* Marc., was found to be completely different in source and sink tissues. *CitSPS1* was expressed to a higher extent in mature leaves and fruits than in young leaves, flowers, and immature fruits. In turn, *CitSPS2* was expressed to a lower level in mature leaves than in flowers and mature fruits, whereas *CitSPS3* was only detected in young and mature leaves^40^. Further, in rice, *OsSPS1* was highly expressed in the youngest fully expanded leaves^41^, while *PpSPS* genes of peach (*Prunus persica* L.) were abundantly expressed in fruits rather than in leaves and phloem-enriched fractions during fruit development^42^. Lastly, in *Cerasus humilis*, *ChSPS1* was expressed to higher levels in fruits than in leaves, and continuously increased during fruit development^27^. The temporal expression of *MeSPS* genes led us to hypothesize a functional collaboration between different *SPS* proteins in the metabolic pathways of cassava.

Recent reports demonstrated that *SPS* may participate in regulating carbon partitioning between starch and sucrose in source leaves^22,43^. For example, in tomato, *SPS* gene expression in transgenic plants revealed a specific role in starch mobilization, whereby diurnal starch content in *NtSPSCi* transgenic leaves was higher than in those of the wildtype^44^. *SPS* activity in cassava leaves was positively correlated with root starch content, implying that starch accumulation in the cassava root might be promoted by *SPS* activity^35,45^. Our results further support these observations. Indeed, we observed that the expression of one or more *MeSPS* genes was significantly and positively correlated with total starch content in the roots during development (Table 3), suggesting that the expression of *MeSPS* genes enhanced starch accumulation in the roots to a certain extent.

In conclusion, our study has expanded our understanding of the molecular bases and differential expression patterns of *SPS* genes, and their correlations with starch accumulation in cassava roots. However, the biological functions of these isoforms and their potential roles in sucrose transport and starch accumulation in cassava warrant further research.

## Materials and methods

### Materials

In the current study, cassava variety Radiation Selection 01(RS01) was used as plant experimental materials, as it is one of the most popular and widely cultivated cultivars in China because of its high root yield and high root starch content. All cassava seed-stems were cultivated at the experimental station of Guangxi University under conventional field management from March to December of 2017 and 2018. The three newest mature leaves (ML), from three healthy cassava plants of the same age, were sampled at 70 days after planting (DAP)—during the seedling stage, in 2017—for *MeSPS* gene cloning. To determine the physiological parameters and relative expression levels of *MeSPS* genes during cassava growth, ML and tubers from three healthy cassava plants were collected in 2 years at the seedling stage (70 DAP, except tubers), at the root formation stage (120 DAP), at the root expansion stage (180 DAP), and at the root maturation stage (210 DAP). Stem samples were also collected at 180 DAP for analysis of gene expression patterns in different cassava tissues in 2017; each stem sampled was separated into three parts (upper/top, middle and lower parts). Tubers were separated into two portions (phloem and xylem parts). All samples were immediately frozen in liquid nitrogen and stored at − 80 °C until used.

### Cloning of *MeSPS *genes from *Manihot esculenta*

Total RNA from cassava leaves was extracted using the Quick Plant RNA Isolation Kit (Huayueyang) according to manufacturer instructions. First-strand cDNA was synthesized using the First Strand cDNA synthesis Kit (Takara), according to the protocol by the manufacturer, and then used for RT-PCR amplification using PrimeSTARMax DNA polymerase (Takara)^46,47^. All specific primers of target genes were designed using the Primer (version 5.0) software for cloning the ORFs based on the complete coding sequence (CDS) of the cassava genome (http://asia.ensembl.org/index.html) (primer sequences were listed in Supplementary Table S3).

The RT-PCR mixture was initially denatured at 94 °C for 5 min, and then ran for 35 cycles of denaturation at 94 °C for 30 s, annealing at 60 °C for 30 s, elongation at 72 °C for 2.3 min with a final 7 min extension step at 72 °C. PCR products were detected by 1.2% agarose gel electrophoresis. Each PCR fragment was separately ligated into the pMD18-T cloning vector (Takara) and then transformed into *E. coli* DH5α competent cells to select positive clones.

### Gene sequences analysis

Amino acid sequences of *MeSPS* genes were predicted by the online ORF finder tool of NCBI (https://www.ncbi.nlm.nih.gov/orffinder). The basic physical and chemical characteristics of these amino acid sequences, including protein length, molecular weight, and predicted isoelectric point, were calculated using the online ProtParam tool (http://www.expasy.org/tools/protparam.html). Multiple-sequence alignment of target amino acid sequences was performed by DNAMAN (version 7.0) software and conserved sequence domains were detected by the Simple Modular Architecture Research Tool (SMART, http://smart.embl-heidelberg.de/) and the InterPro database (http://www.ebi.ac.uk/interpro/search/sequence/)^48^. Analysis of exon–intron structures was visualized by the Gene Structure Display Server (GSDS) 2.0 program (http://gsds.cbi.pku.edu.cn/). Conserved motifs of *MeSUS* protein were analysed by the MEME program (http://meme-suite.org/tools/meme) and TBtools v0.6, http://www.tbtools.com/) software, according to the method of Bailey et al.^49^. The maximum number of motifs was set to 12, all other default parameters were normal. Chromosomal locations and coordinates of the *MeSUS* genes were mapped by the MG2C v2.0 program (http://mg2c.iask.in/mg2c_v2.0/) based on genomic information available from the cassava genome database (http://www.phytozome.net/cassava). The ClustalW algorithm was used to align the target protein sequences, and MEGA vX.0 software (https://www.megasoftware.net/), with the neighbour-joining (NJ) approach and 1000-replication bootstrap methods^50^, was used to construct the phylogenetic tree.

### RT-qPCR analysis

Total RNA was extracted from each sample from each growth stages according to the protocol by the manufacturer (Huayueyang). The first-strand cDNA was synthesized using the instruction manual of First-Strand cDNA Synthesis Kit (Vazyme). Gene-specific primers for RT-qPCR were designed using the Primer 5.0 software (primer sequences were summed in Supplementary Table S3), using the housekeeping gene TBP-associated factor 15B (TAF15b) as an internal control for relative gene expression analysis^51^. The RT-qPCR reaction system contained 10 μl 2 × SYBR qPCR MasterMix (Vazyme), 2 μl template cDNA, 0.4 μl primers, and was made up to 20 μl with ddH_2_O. The amplification process was performed using the following protocol: 95 °C for 3 min, followed by 45 cycles at 95 °C for 10 s and 60 °C for 30 s. Three biological replicates were measured per reaction. Relative expression levels of the genes were calculated by the 2^−ΔΔCt^ method. The error bar for each expression level was calculated based on the biological replicates using Microsoft Excel 2019.

### Determination and correlation analysis of sucrose, starch contents and enzymatic activity

Sucrose and total starch contents were analysed according to the method described by Huang et al*.*^52^, while *SPS* activity was measured following the instruction manual of the Plant Enzyme-linked Immunosorbent Assay (ELISA) Kit (Jianglai). Three biological replicates were measured per sample.

Statistical analyses were performed using Pearson algorithm of IBM SPSS 19.0 (SPSS Science, Chicago, IL, USA) software. Correlation analysis was conducted between *SPS* activity, sucrose content, total starch content, and *MeSPS* expression levels at different developmental stages, in 2017, and 2018.

## Supplementary information

Supplementary information.

Supplementary tables.

## Data Availability

All data generated or analysed during this study are included in this article and its supplementary information files.
