# Supplementary material for: Molecular cloning and expression analysis of sucrose phosphate synthase genes in cassava (Manihot esculenta Crantz)
Source: Sci Rep. 2020 Nov 26;10:20707. doi: 10.1038/s41598-020-77669-9 (PMC7692556; doi:10.1038/s41598-020-77669-9)
Supplement: Supplementary file 1 — Supplementary information. [file 41598_2020_77669_MOESM1_ESM.pdf]

Molecular cloning and expression analysis of sucrose phosphate synthase genes in cassava (*Manihot esculenta* Crantz)

Tangwei Huang[1], Xinglu Luo[1,2], Maogui Wei[1], Zhongying Shan[1], Yanmei Zhu[1], Yanni Yang[1] & Zhupeng Fan[1]

[1]College of Agriculture, Guangxi University, Nanning 530004, China
[2]State Key Laboratory for Conservation and Utilization of Subtropical Agro–bioresources, Nanning 530004, China
Correspondence and requests for materials should be addressed to X.L. (email: luoxinglu@sina.com)

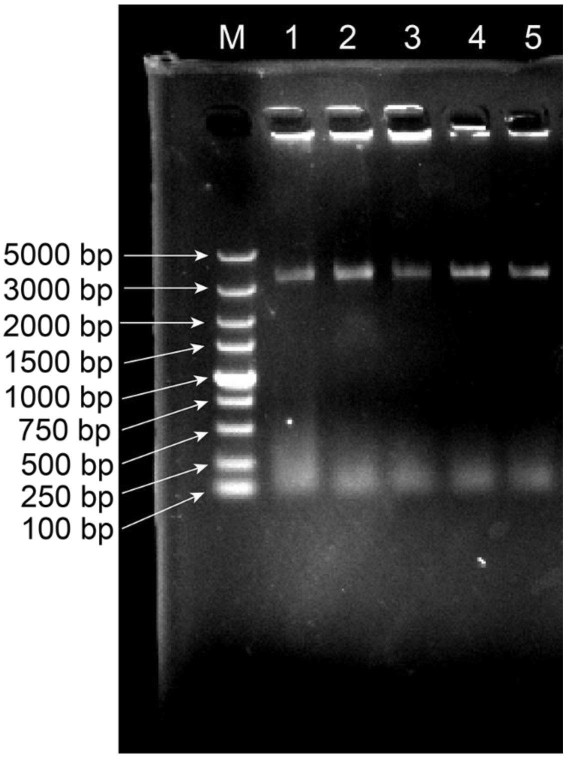

Supplementary Figure S1. The RT–PCR amplification of *MeSPS* genes. Lane M represents the 5000 bp DNA marker. Lanes 1–5 indicate RT–PCR products of *MeSPS1*, *MeSPS2*, *MeSPS3*, *MeSPS4*, and *MeSPS5*, respectively.

Supplementary Data S1. Nucleotide sequences of each *MeSPS* gene.

> ***MeSPS1***

TGTTCTGTAATTTTAGTATGTTAAAACGAAAAGGAAAGTTTTTTTTTTTAAA
AAAAAAAAATAAAGAATAAGCAAAGAAAGCACGAGACTGTTATAAGAAGA
AGTTGGAGTAGTAAACTCTCCCCATTTCCAGCCTCTAATCTCTCTGCCTT
TGTCTTTTTTCTTCGATATTCATTCACCAGCTTACATTTACAAGCAAAAAC
CACTGCTCTAATCACAATTCTCCCTTTCTCCATTAGCTGATTCTGGTGGA
GGAGGAGCAAATCAACTAATGGCTGGCAACGATTGGATTAACAGCTACC
TCGAGGCGATACTCGATGTCGGCCCCGGCCTGGATGATGCTAAGTCGT
CTTTGTTGCTCAGGGAAAGAGGAAGGTTTAGTCCCACTCGCTATTTCGTC
GAAGAAGTCATCACTGGATTTGATGAGACTGATCTCCATCGCTCATGGGT
TAAGGCTCAGGCAACGAGGAGTCCGCAAGAGAGGAACACCAGATTGGA
GAATATGTGCTGGAGGATTTGGAACTTAGCTCGCCAAAAGAAGCAGCTT
GAGGGGGAACTTGCCCAGAGGAAAGCTAAACGACATCTTGAAAGGGAG
AAAGGCCGGAGAGAAGCAACTGCTGACATGTCTGAAGATCTATCAGAGG
GAGAAAAAGGAGATGCAGCAGGTGATGTATCGGTCCATGGTGATAGTAA
CAGAGGCAGACTGCCTAGGATCAATTCTGTTGATGCAATGGAGGCATGG
GCTAATCAGCAGAAGGGCAAAAAGCTGTACATTGTGTTAATAAGCCTTCA
TGGTCTAATACGAGGTGAAAATATGGAGCTTGGGCGGGATTCTGATACG
GGTGGTCAGGTTAAATATGTTGTGGAACTTGCAAGAGCTTTAGGCTCAAT
GCCAGGAGTCTATCGAGTGGACTTGCTAACTAGACAAGTATCAGCACCA
GATGTAGATTGGAGTTATGGTGAGCCCACAGAGATGCTGACTCTTAGAA
ACTCAGAAGATTTTGAGGATGAGATGGGGGAGAGCAGTGGTGCTTATAT
TGTTCGTATACCATTTGGACCAAAGGATAAATACATTCCTAAAGAACATTT
GTGGCCTCACATCCCCGAATTTGTTGATGGTGCACTTAACCACATAATAC
AGATGTCCAAAGTTCTGGGGGAGCAAATTGGTGGTGGGAAGCCAATCTG
GCCTGTTGCCATCCATGGACATTATGCGGACGCAGGCGATTCTGCTGCA
CTTCTATCTGGTGCTTTAAATGTGCCAATGCTTTTTACTGGCCACTCACTT
GGCCGAGATAAGTTAGAACAGTTACTAAAACAAGGCCGACTATCAAGGG
ATGAAATAAATTCTACGTACAAAATAATGCGTCGGATAGAGGCTGAGGAA
TTCTCCCTGGATTCTTCTGAAATAGTCATAACTAGCACTAGACAGGAGAT
TGATGAGCAGTGGCGCTTATATGATGGTTTTGATCCAATATTGGAGCGTA
AACTGCGAGCAAGAATCAAGCGAAATGTGAGCTGTTATGGAAGATCCAT
GCCTCGCATGGCTATAATTCCTCCTGGAATGGAGTTTCATCACATTGTTC
CCCAAGAAGGGGATATGGATGGTGAATTGGAAGGAAATGAAGACCATCC
TACTTCTCCTGATCCACCAATATGGAACGAGATAATGCGCTTCTTTACAA
ATCCTCGGAAGCCTATGATTCTTGCCCTTGCCCGGCCAGATCCTAAAAA
GAACATTACAACTTTGGTTAAAGCATTTGGAGAATGTCGTCATTTGAGAG
AGCTCGCTAATCTTACGCTAATTATGGGTAACCGAGATGGAATTGATGAA
ATGTCAAGCACAAATGCTTCTGTCCTACTTTCAGTGCTTAAGCTTATTGAC

AAGCATGATTTGTATGGGCAAGTTGCATACCCTAAACACCACAAGCAGG
CTGATGTCCCTGACATATATCGCCTAGCAGCAAAGACAAAGGGGGTTTT
CATCAATCCAGCTTTCATTGAGCCGTTTGGGCTTACTTTGATTGAGGCAG
CAGCTCATGGTTTGCCCATTGTTGCCACAAAAAATGGAGGTCCTGTAGAT
ATACACCGGGTACTTGACAATGGTCTTCTTGTTGATCCTCATGATCAACA
GTCTATTGCTGATGCTCTTCTAAAGCTTGTTGCTGACAAACAGCTTTGGG
CAAAATGCCGACAAAATGGATTGAAGAATATTCACCTATTTTCATGGCCA
GAGCATTGCAAGACTTACCTATCACGAATAGCCAGCTGCAAACCAAGGC
ATCCACAGTGGCAAAAGGATAATGATGGAAATGATTCTTCAGACACGGAT
TCACCAGGTGATTCCTTGAGGGATCTGCATGATATATCTTTGAACTTGAA
GTTCTCATTGGATGGAGAAAAGACTGGAGCTAGTGGAAATGATAATTCTA
TAGAATCTGAAGGAGATTCTGCTGATAGAAAGGGCAAGTTAGAGAATGC
TGTTTTGGCATGGTCAAAGGGTGTTTTGAAGAACACACAAAAGACTGGAT
ACATAGATAAAGGAGAACAAAACAACAGTTCTGGTAAGTTTCCAGCTTTG
AGGAGGAGGAAACAAATTTTTGTTATTGCAGTAGATTTCGATACAATTTCT
GGGCTTATAGAGGCAACAAAGAAAATTTTTGATGCCGTTGAAAGAGAAAG
GACAGAAGGCTCAATAGGGTTCATATTATCAACGTCTTTGACCATGTCTG
AAATAAACTCATTTCTGGTCACAGGGGGGTTTAGCCCCAGTGATTTTGAT
GCTTTTATATGCAACAGCGGAAGCGAACTCTATTACTCAAATCTAAATGC
AGAGGATAATCCCTTTGTGGTTGACTTTTATTACCACTCACATATTGAGTA
CCGATGGGGTGGAGAAGGGCTTAGGAAGACTTTGGTTCGCTGGGTTTCT
TCAGTTATCGATAAGAAGGCTGAGAAAGGGGAACATATTGTTATGGCAG
CTGAACAGCTATCAACCAACTATTGTTATGCTTTCAAAGTGCAAAAGCCA
GGAATGGTTCCCCCAGTTAAGGAACTCCGGAAGTTGCTAAGGATTCAGG
CTCTCAGATGCCATGTTATATATTGCCAAGATGGGACCAGGATAAACGTA
ATTCCAGTATTGGCTTCTCGCTCCCAAGCCCTAAGGTATCTTTATGTCCG
ATGGGGGATCGAATTGGCAAATGTGGTGGTTTTTGTTGGAGAATCTGGG
GATACAGATTACGAAGGATTGCTTGGTGGGCTACACAAGAGCGTAATATT
AGGGGGAGTTTGTACTAGTGCAAGTAGCCAACTCCATGCCAACAGAAAC
TACCCGCTCGCAGACGTCATAGCATCAGACAGCCCCAATATCGTTCGCA
CAACTGAAGAATGCACCAGTTCCGATATCCGGGCCTCGTTGAAGCAATT
AGCATGTGTCAAGGGATAGACAGTCATACCCTTACCCTTCATCATTTCAA
ATTTGTTCTAACCTTGTCTGTGTTGCTTTCATCATAAGGAGGACCTGGTT
GCTTTCATGCCAAGATCTCTTGTTACTCCAATGGCATTAGCGCATCTATTT
TTCTATCCTGAAGAAGCTTGCTTGTGTGTTTTTTCCCTGGAGACTCGAAC
GCCAACTTACTTTCTACTTTTTCCAATTTGTTTATTCCCATGTAGAGTCGA
TCCTTGTGTGCTTTATTGGCCAGCAAGTTTCTAATTGAAGCACTATGTTG
CTTTCTGTCCTATATCTGTGGCTGTTGTAGAAAGTTACATGTAGTTTTGGC
ATGTATCAATCAGACCAGTCAAGCCCAAAATAATACAAAATATCATATCAG
AATGTTTACAAAAAAAAAAAAAAAAAA

> ***MeSPS2***

ATGTCTGAAGACTTATCAGAAGGAGAGCGAGGAGATGTGCATGGTGAAA
TATTGTCTCATGGTGGGAGTGTTAGAGGAAGAATGCCTAGAATAAGTTCT
GTTGACGTCATGGAGAATTGGGCTAAACAACACAAGGAGAAGAAACTAT
ATATCGTGCTGATAAGCCTTCATGGCTTGATACGTGGTGAAAACATGGAG
CTTGGGCGAGATTCTGATACTGGTGGCCAGGTTAAGTATGTAGTAGAAC
TTGCAAGGGCCTTAGGCACAACGCCAGGGGTTTATCGGGTCGATTTGCT
GACAAGACAAGTCTCAGCTCCAGATGTGGATTGGAGTTACGCTGAACCT
ACAGAGATGCTGAATCTGATAAATTCTGAGAATTCTATGCAGGAGCATGG
GGAAAGCAGTGGTGCCTATATAATTCGTATACCATTTGGCCCAAAGGATA
AATATATCCAAAAAGAACTCCTTTGGCCTTACATTCCAGAATTTGTGGATG
GTGCACTTAACCATATTATCCAGATGTCTAGAGTTTTAGGGGAGCAAATT
GGCAGTGGAAATCCAGTCTGGCCTGTTGCAATTCATGGTCATTATGCTG
ATGCAGGGGACTCTGCTGCTCTGCTCTCTGGAGCTCTAAATGTTCCTAT
GGTTTTTACTGGGCATTCACTTGGACGCGATAAGCTTGAACAACTTTTGA
AACAAGGAAGGCAATCAAGAGAAGAAATAAATGCAACCTATAAAATAATG
CGAAGAATAGAGGCAGAAGAGTTAACTCTTGATGCTTCTGAGATTATTAT
AACTAGCACTAGACAGGAGATAGAAGAACAATGGCGCCTGTATGATGGT
TTTGATCCTGTACTAGAGCGCAAACTCAGAGCAAGGATCAAGAGGGGAG
TAAGCTGTCATGGCAGGTTTATGCCTCGCATGATTGTGATACCTCCTGGA
ATGGAATTTCATCATATTATTCCACATGATGCGGATATGGATGGGGAAGA
TGAAGAAAAATTTGAAGACTCCCCTGCTGCTCCTGATCCACCAATTTGGT
CGGAGATAATGCGTTTCTTTTCCAATCCTCGGAAACCCATGATACTTGCC
CTTGCCAGGCCTGACCCCAAAAAGAATATAACCACTTTAGTCAAAGCATT
TGGAGAATGCCGCCCTCTAAGGGAGCTTGCTAACCTTACATTAATAATGG
GAAACCGCGATGATATCGATGAAATGTCAAGCACAAATGCTAGTTATCTC
CTTTCCATTATTAAGCTCATTGATAAATATGATCTGTATGGCCACGTAGCA
TATCCTAAACACCACAAGCAGTCTGATGTTCCTAACATTTATCGCCTTGC
AGCAAAAACAAAGGGGGTTTTCATCAATCCTGCTTTTATCGAGCCCTTTG
GGCTTACGTTGATTGAGGCTGCGGCTCATGGTTTGCCTATTGTTGCTACA
AAAAATGGGGGTCCTGTTGACATTCATCGGGTACTTGACAATGGATTACT
AGTTGATCCCCACGACCAGCAGTCTATTGCTGATGCTCTTCTCAAGCTTG
TTTCAGATAAACAACTTTGGGCAAGATGCAGGCAGAACGGATTGAAAAAT
ATTCACCTTTTCTCATGGCCAGAGCATTGTAAGACATACTTGGCTCGAAT
AGCCAGTTGCAGGCCTAGGCAGCCCCAGTGGCAGAGAAGTGAAGATGG
GTTGAGAAATGAAGAGTCCGATTCACCTGGTGATTCTTTAAGAGACATTA
AAGATTTATCTTTGAAGCTTTCATTGGATGGAGACAAAAATGAAAGTGGA
AATCTTGATAATTCTCTTGATACAGAAGAAAATGCTTCAGATAGGAAGAAT
GTGTTAGGCAATAATGCTTTGACATTGTCAAAAGATGCTATAAGAGGTGC
ACAAAATGAATCAATAGAGAAGGCAGACAATAATATTTGCAGCAGCAAAT
TTCCATCACCGAGGAAGAGGAAATATATCTTTGTTATTGCAGTAGATGGT

GATACGACTTCAGATTCTCTTGAAGCTATTAAGATAGTTGTTGAGGTAGG
AATGAAGGAAACCTCTATAGGTTTCATTTTATCGACTTCCATGACCATATC
TGAGGTGTACTCTCTTCTGGCCTCAGGAGGCTTGAGCCCATTGGATTTT
GATGCATTTATATGTAATAGTGGTAGTGAAGTCTATTATCCATCTTCAAGC
AATGGGGATACTACTGGCCTTCCTTTTGTATTAGACCTGGATTATCATTC
ACACATTGAATACCATTGGGGTGGAGAAGGTTTGAGGAGGACTCTGGTT
CGTTGGGCTGCTTCTATCAATGATAAGCATGGACAGGAACAAATTGTTGT
GGAGGATGAATCAGGATCAACAGCTCATTGCTGTGCATTTAAAGTGAAA
GAACCATCAATGATCCCTTCATTTAGAGAACTTCGGAAACTGATGAGAAT
TCAGGGCCTCCGCGGCCATGTTATTTATTGTCAGAATGGTTCCAAGATAA
ATGTGATCCCTGTGTTGGCTTCTCGATCCCAAGCTCTAAGGTATCTGTAT
ATTCGGTGGGGTGCAGACTTGTCAAAATTTGTGATTTTTGTGGGGGAATG
TGGTGATACAGATTACGAAGGCTTACTTGGTGGAATTCACAAGTCAGTTA
TATTGAAGGGAGTTGGCAGCAATGCTCGTAAGCTTCATGCCGACAGAAG
CTATCCTCTGGAAGATGTTACACCATTTGATAGTCCCAATGTTGTCCAGA
GTGAGGGATGCAACGTTAATGACATAAAGCAATCACTTGAGAAACTGGG
GGTTCTCAAGGTCTAG

> ***MeSPS3***
ATGGCCGGAAACGAATGGATTAATGGGTACCTGGAGGCGATACTGGATA
GTGGGGCGGGTGCTATTGAAGAGCATAAGCCGGCGCCGCCTGTAAATC
TGAGCGACAGGGGGCATTTTAATCCGACTAAGTACTTTGTGGAGGAGGT
GGTCACTGGAGTCGATGAGACTGATCTGCACAGGACGTGGATTAAGGTG
GTTGCCACCCGCAACACCCGCGAGCGAAGCTCCAGGCTCGAGAACATG
TGCTGGCGTATTTGGCACCTCACTCGCAAGAAGAAGCAGTTGGAATGGG
AGGGACAACAAAGACAGGCAAGTCGAAGATGGGAACGGGAAGAAGGAA
GAAGGGACGCAACCGAAGATCTGTCAGAGGACTTGTCAGAAGGAGAGA
AAGGGGATATCGTGGGAGAGATGTTGCTAAGTGAGACCCCAAGGAAAAA
ATTCCAGAGAAACTTTTCCAACTTGGAAGTTTGGTCGGAGGACAAAAATG
AAAAGAAACTCTACATTGTCCTTATCAGTTTACATGGTTTGGTACGTGGA
GAAAATATGGAGCTTGGTCGAGATTCTGACACTGGTGGACAGGTCAAAT
ATGTGGTAGAACTTGCTAGAGCACTTGCTAGAATGCCTGGGGTGTATAG
GGTAGACCTTTTCACTCGCCAAGTTTCCTCTCATGAAGTTGACTGGAGTT
ATGGTGAGCCAACAGAGATGCTAACTTCAGGATCTGAAGATGCTGATGG
CAATGAGGTTGGAGAAAGCAGTGGGGCATATATTATTAGGATTCCCTTTG
GTCCCCGTGATAAGTACCTCAGGAAAGAATTACTTTGGCCCTATATTCAG
GAATTTGTGGATGGGGCTCTTGCTCATATTCTTAATATGTCAAAAGTTTTG
GGTGAGCAGATTGGAGGGGATGGCCCTGTGTGGCCATATGTCATTCATG
GTCATTATGCTGATGCAGGCGATAGTGCTGCACTTTTATCAGGTGCTTTA
AATGTGCCAATGGTATTAACAGGACATTCACTTGGAAGAAACAAGCTTGA
ACAGCTTCTTAAGCAGGGTCGGCAATCAAAGGAAGACATCAATTCAACAT

ATAAAATAATGAGGAGGATAGAAGCAGAAGAGCTCGCACTTGATGCTGC
TGAACTAGTGATCACAAGTACCAAACAGGAGATTGAAGAGCAATGGGGA
CTTTATGATGGATTTGATGTGAAGCTTGAAAAGGTTTTGCGTGCTCGCTC
TAGAAGAGGTGTCAATTGCCATGGCCGTTACATGCCAAGGATGGTGGTT
ATCACTCCTGGTATGGACTTCAGCAATGTTGTGGTTCAAGAAGATGCTCC
AGAAGTTGATGGGGAACTTGCATCACTTATTGGTGGTTCTGATGGTTCCC
CAAAAGCAATCCCTGCAATATGGTCTGAAGTGATGAGATTCCTTACTAAT
CCTCACAAGCCAATGATTTTGGCCTTGTCAAGGCCTGACCCAAAGAAAAA
TATTACTACGTTACTGAAAGCCTTTGGAGAGTGCCGTCCATTAAGAGAGC
TTGCCAATCTCACACTAATCATGGGAAACAGGGACGATATAGATGAGATG
ACAGGTGGAAATGCAAGTGTTCTCATGACAGTATTGAAACTGATAGACAA
GTATGATCTCTATGGGCTAGTTGCATATCCAAAGCATCACAAGCAATACG
AAGTTCCAGATATTTATCGGCTTGCTGCAAAGACAAAGGGAGTTTTCATA
AACCCAGCATTGGTCGAGCCTTTTGGCCTTACCTTAATTGAGGCTGCTG
CACACGGGCTTCCTATGGTGGCAACTAAAAATGGTGGCCCAGTTGACAT
CAATCGAGCATTGAACAACGGTCTACTTGTGGATCCTCATGATCAGCAAG
CTATTGCTGATGCACTACTTACATTGGTATCAGAGAAGAATCTATGGCAT
GAGTGCAGAAAGAATGGTTGGAAAAATATACACCTTTTCTCATGGCCAGA
ACACTGCCGCACTTACTTGACTAGGGTAGCAGCATGCCGAATGAGGCAC
CCACAGTGGCAAACTGATACTCCAGGAGATGAGATGGCTGCTGAAGAGT
CATCTCTCAATGATTCCCTGAAGGATGTGCAAGATATGTCTCTTAGGCTC
TCAATTGATGGCGATAAATCTTCATTAAATGAATCACTCGACTATTCAGCT
GCAGCTGCAGGTGACCCGGAGATACAAGCTCAAGTAAACCAGGTAATGA
GGAAGATCAAGAAACCAGAGGCTGGCCCAAAAGATGCTGAAGGTGGAA
AGAATGAGACTGGCATGAGCAAGTATCCCATGTTGAGGAGGCGACGAAG
GTTGATTGTAATAGCTCTTGACTGCTATGGCGCTGAAGGGGCTCCTGAA
AATAAGATGATTCAAGTTGTGCAATATGTAATTAAAGCTGTTAGGTCAGA
CTCACTATTTGCAAGAACATCAGGGATTGCTTTAGCTACAGCTATGCCAT
TGTCAGAAACAGTAGAATTCTTGGCATCGGCAAAGATTCAAGTAAATGAG
TTTGATGCATTGATTTGTAGCAGTGGTAGTGAATTGTATTACCCTGGAAC
TTATACTGAAGAGAATGGAGAACTTTTACCGGATCCAGATTATGCATCAC
ATATAGACTACCGCTGGGGTTGTGAAGGTCTTAAAAAAACCATCTGGAAG
CTAATAAACACAACTGAAGGTGCAGAACAATCTAAAGGATCATCCAGCCT
CATCGAGTTAGATTCAAAATCAAGCAATGCCCATTGCGTCGCATACTGGA
TCAAGGATCGCAAAAAGGTTATGAAAGTGCATGATTTGAGGCAAAAACTA
AGGATGCGGGGTCTTCGTTGTCATCCAATGTACTGCAGGAGCTCAACAA
GGATGCAAATCATTCCTCTCTTGGCTTCTCGAGCACAGGCGCTGAGGTA
CCTTTTCGTCCGTTGGAGGTTGAATGTTGCAAACATGTATGTAATTCTTG
GTGAAACTGGAGACACTGACTATGAGCAAATGGTAGCTGGGGCTCATAA
GACCATAATAATGAAAGGTGTGGTGATAAAAGGATCTGAAGAATTGCTAA
GATCCATGGACTTAAAAGATGACTTTGTTCCTAAAGAGAGTCGCTTGATT

GCACATTTGAGTGGTGAATCATCAGCCAGTGAGATCGCTGAAGCTTTGA
AGCAAGTATCAAAAGCTACTGGGATGTGA

> ***MeSPS4***

ATGGCTGGAAATGATTGGATAAATGGGTACTTGGAAGCAATTCTAGATGT
GGGAAGTAGTCTAAGGAAAAGAAATGATGGGCAGTTGAAGATTTCTAAG
TTTGAAGACAGCAAACAAAAGGAAGACAAGTCTTTTAGTCCTACTAAGTA
CTTTGTTGAAGAAGTTATTAATAGCTTTGATGAATCTGATCTCCACAGGAC
ATGGGTCAAGGTGATAGCAACAAGGAATACTCGTGAACGCAGTAACAGG
CTTGAGAATATGTGCTGGAGGATTTGGCATCTTGCCCGTATGAAGAAAAA
GATAGAATGGGATGATGCACAAAGACTTGCCAGAAGACGGCTGGAGAGA
GAGCAAGGGCGTAATGATGCTGCTGATGACCTTTCTGAGCTATCCGAAG
GGGAGAAGGAAAAGGGTGACACCAACCTTTCAGAACCTGTCAAGAACTT
CAGCAGAATTAACTCAGATATGCAAATATGGTCTGATGAAGAAAAACCAA
GGCGCCTATACATTGTGCTTATCAGTATGCATGGTCTGGTGCGTGGAGA
GAATATGGAACTTGGAAGAGATTCTGATACTGGGGGTCAGGTAAAATAT
GTTGTTGAACTTGCTCGAGCTCTAGCCAATACAAAAGGAGTCTATAGAGT
AGATCTCTTGACCAGACAAATCAGCTCCCCTGAGGTAGACTACAGCTAT
GGTGAGCCCATTGAGATGCTGGCATGCCCACCTGATGGAAGTGGCAGTT
GTGGAGCCTACATAGTCCGCATTCCTTGTGGTCCCCGCGAGAAGTACAT
ACCAAAAGAATCACTCTGGCCTTACATACCTGAATTTGTAGATGGAGCCT
TAAGCCATATTGTGAACATGGCTAGAGCTCTAGGGGAACAAGTTAATGG
GGGGAAGCCAACTTGGCCATATGTAATTCATGGCCACTACGCTGATGCT
GGGGAGGTGGCATCACATTTGTCCGGGGCCTTGAATGTGCCAATGGTG
CTAACAGGACACTCTCTGGGGAGGAACAAGTTTGAGCAATTGCTTAAGC
AAGGAAGGCTTTCTAAGGAAGACATAAACGTGACATACAAGATAATGAG
GAGGATTGAAGCAGAAGAGTTGGGGTTGGATGCAACTGAGATGGTGGTA
ACTAGCACAAAGCAAGAGATTGAAGAGCAATGGGGCTTGTACGATGGGT
TTGATATCAAGTTGGAAAGAAAGCTGAGGGTTAGAAGACGCAGAGGAGT
AAGTTGCATGGGAAGACACATGCCAAGGATGGTGGTTATACCCCCAGGC
ATGGACTTCAGTTATGTGACAACACATGATTCATTGGAAGGTGATCTCAA
GTCATTAATTGGGCCTGACAGAACTCAAACTAAAAGGAATCTGCCTCCAA
TATGGTCCGAGGTAATGCGGTTTTTCACAAATCCTCACAAGCCTACCATA
CTTGCATTGTCCCGTCCTGACCCAAAGAAAAATGTCACCACATTACTCAA
GGCATTTGGGGAGTGCCAGCGCCTCCGTGAGCTAGCCAATCTGACTCTA
ATACTTGGTAACAGAGATGACATTGAAGAGATGTCTAACAGTAGCTCAGT
TGTTCTTACAACTGTGCTTAAGCTCATTGACAAGTATGATTTGTATGGTCA
AGTTGCTTACCCCAAGCATCATAAGCAATCTGAAGTTCCTGACATTTACC
GTCTGGCTGCAAAAACAAAGGGAGTTTTCATTAATCCAGCTCTGGTGGAA
CCATTTGGTCTCACACTCATAGAGGCAGCTGCTTACGGTTTACCTGTCGT
TGCTACCAAAAATGGTGGACCAGTGGACATTTTAAAGGCTCTTAACAATG

GCCTACTGGTGGATCCTCATGATCAGAAAGCAATAGCAGATGCACTTCT
GAAGCTTGTTGCTGACAAAAACCTCTGGACTGAATGCCGAAAAAATGGC
TTAAAAAACATTCATCGCTTTTCTTGGCCAGAACATTGCCGTAATTATCTC
TCACACATTGAACACTGCAGGAACCGCCACCCAACAAGCCGTCTTGAGA
TCACACCGGTTCCAGAAGAGCCAATGAGTGACTCTCTAAAGGATGTGGA
GGACCTGTCTTTGAGATTCTCTATAGAAGGCGATCCCAAGCTTAATGGAG
AGCTAGATGCAACAACTAGACAAAAGAAACTAATTGAAGCCATTACCCAA
GCTGCTTCATTCAATGGTAACACAAATGTTACATATAGTCCTGGCAGAAG
GCAGATGCTATTTGTGATAGCTGTTGATTGTTATGACTGTAATGGGAAAA
GCACCGAGACCTTCCAAGAAATTATCAAGAATGTGATGAAAGCTGCTGG
ATCATGCCTAGGGTTGGGCAGGATAGGCTTTGTACTGTCAACCGGCTCA
AGTTTACAAGAGACAATGGAAGCTTTAAGATATTGCCCAGTGAATATAGA
AGATTTTGACGCGATAATATGTAATAGTGGAAGTGAGATGTACTGCCCAT
GGAGAGACATGGTGGCTGATTTGGACTATGAAGCCCATGTGGGGTATAG
GTGGCCTGGTGAGAATGTTAGGTCTATGGCAATTAGGCTAGCTAAGGTA
GAAGATGGGGCTGAAGATGACGTACTCGAGTATGTTCAAGCAAGTGGTT
CTAGGAGCTATTCATATATCATCAAACCAGGAGCCAAGACTCGAAAGGTT
GATGAAATCCGACAAAGGCTCCGAATGAGAGGCATTCGATGCAGCCTTG
TCTACACACGTGCAGCATCAAGGTTAAATGTGATTCCATTATTTGCATCA
AGGAAACAAGCCCTCAGGTATCTTTCAGTTAGGTGGGGAATCGATCTTTC
CAAAATAGTAGTCTTTGTTGGAGAAAAAGGGGATACAGATTATGAAGAAC
TACTAGCTGGCCTCCATAAGACCCTTATTATGAGAGGTTCTGTGGAGTAT
GGAAGTGAGAATCTTCTTTGCGGCCAAGACGGCTTTAAAAGGGAAGATA
TTATCCCCCAAGAAAGCCCTAGCCTACGCTTTGTGGAGGAAAATTATGAA
AACCTGTCTACAGCTTTAGAGACTCTAGGGATCAAGTGA

> ***MeSPS5***

ATGGCAGCAAATGATTGGATAAACGGGTACTTGGAAGCTATTTTAGATGT
GGGAAGTAGTCTAAGGAAAAGAAATGAAGGCAAGTTGAATGTTGTTAAGT
TTGAAGACAGCAAGGAAAAGGAAGACAAGTCTTTTAGTCCAACTAAGTAC
TTTGTGGAAGAAGTTATCAATAGCTTTGATGAGTCTGATCTTCACAGGAC
ATGGGTCAAGGTGATAGCAACAAGGAATACTCGTGAACGGAGTAACAGG
CTTGAGAATATGTGCTGGAGAATTTGGCATCTTGCCCGTGAAAAGAAAAA
GATAGCATGGGATGATGCACAAAGGCTTGCCAGAAGACAGTTGGAGCTA
GAGCAAGGGCGTAATGATGCTGCAGACGATCTTTCTGAGCTATCCGAGG
GAGAGAAGGAAAAGGGTGATGCCAACTTCTCAGAACCTGTCAAGCATTT
CAGCAGAATTAACTCAGATATGCAAATATGGTCTGATGATGAAAAGCCTA
GGCACCTGTACATTGTGCTAATCAGTATACATGGTCTGGTGCGTGGAGA
AAATATGGAACTTGGAAGAGATTCTGATACAGGTGGTCAGGTAAAATATG
TTGTTGAACTTGCTCGAGCTCTAGCAACTACAAAAGGAGTCTATCGAGTG
GATCTCTTGACCAGACAAATAAGTTCCCCTGATGTGGACTTTAGCTATGG

TGAACCCATTGAGATGCTGTCATGCCCACCCGATGGCTGTGGAAGTTGT
GGAGCTTACATTGTCCGCATTCCTTGCGGTCCCCGTGACAAGTACATAC
CAAAAGAATCACTCTGGCCTTACATACCCGAGTTTGTTGATGGGGCTTTA
AGCCATATTATGAACATGGCTAAAGCTCTAGGGGAACAAGTTAATGGGG
GAAAGCCTACTTGGCCATATGTAATTCACGGCCACTATGCTGATGCTGG
GGAGGTGGCGTCACATTTGTCCGGGGCCTTGAATGTGCCCATGGTGCTA
ACAGGACACTCTCTGGGGAGGAACAAGTTTGAGCAATTACTTAAACAAG
GAAGGCTTTCTAGGGAAGACATTAATGCAACCTACAAGATAATGAGAAG
GATTGAAGCAGAAGAGTTGGGGTTGGATGCATCTGAGATGGTGGTGACC
AGCACAAAGCAAGAGATTGAAGAGCAATGGGGCTTGTATGATGGGTTTG
ATCTGCAGTTGGAAAGAAAGCTTAGGGTTAGGAGGCGCCGAGGAGTAA
GTTGCATGGGAAGACACATGGCGAGAATGGTGGTTATACCTCCTGGCAT
GGACTTCAGCTATGTAACAACACAAGATTCATTGGAAGGTGATCTCAAGT
CATTAATTGGCTCTGACAGAACTCAAACTAAAAGGAATCTGCCTCCAATA
TGGTCCGAGATAATGCGCTTTTTCACAAACCCTCATAAGCCTACTATACT
TGCATTGTCCCGTCCTGACCCAAAGAAAAATGTCACCACATTACTCAAGG
CTTTTGGGGAGTGCCAGCGCCTCCGAGAGCTAGCCAATCTGACTCTAAT
ACTTGGTAACAGAGACGACATTGAAGAGATGTCTAACAGTAGCTCAGTTG
TTCTCACAGCTGTGCTCAAGCTCATTGACAAGTACGATTTGTATGGTCAA
GTTGCTTACCCCAAGCATCACAAGCAAACTGAAGTTCCTGATATTTATCG
TCTGGCTGCAAAAACAAAGGGAGTTTTTATTAATCCTGCTCTGGTGGAAC
CATTTGGTCTCACACTTATAGAGGCGGCTGCTTATGGTTTACCTGTTGTT
GCTACCAAAAATGGTGGACCGGTGGACATTTTGAAGGCTCTTAACAATG
GCCTATTAGTAGACCCTCACGATCAAAAAGCAATAGCTGATGCACTTCTA
AAGCTTGTTGCTGACAAAAACCTCTGGACAGAATGCCGAAAAAATGGCTT
GAAAAACATTCACCGCTTTTCTTGGCCAGAACATTGTCGTAACTACCTCT
CCCACATTGAACACTGCAGGAACCGCCATCCAACAAGCCGTCTTGAGAT
CACATCAATCCCTGAAGAGCCAATGAGTGACTCTCTAAAGGATGTGGAA
GACCTGTCTTTGAGATTCTCTATAGAAGGAGAAAACAAATTTAACGGAGA
GCTAGATGCAGCAACTAGACAAAAGAAACTAATTGAAGCCATTAGCCAAG
CAACTTCATCCAATGGTAATGCAAGTGTTACTTATAGTCCAGGTAGGAGG
CAGATGCTATTCGTGATAGCTGTTGATTGTTATGACTGTAACGGAAAAAG
CACAGAGGCCTTCCAAGAAATTACCAAAAATGTGATGAAAGCTGCAGGA
TTATGCGTAGGATTAGGCAAAATAGGTTTTATACTGTTAACTGGCTCAAG
TTTACAAGAGACAATGGAAGCTTTAAGATGTTGCCCTGTGAATATAGAAG
ACTTTGATGCAATAATATGTAATAGTGGAAGTGAGATGTACTATCCATGG
AGGGACATGGTGGCTGATTTGGACTATGAAGCCCATGTGGATCACAGGT
GGCCTGGTGAAAATGTTAGGTCTGTGGCAATTAGGCTAGCAAAGATAGA
AGATGGGGCTGAAGATGATGTTCTTGAATATTTTCAGGCATGTGGTTCTA
GGTGCTATTCTTACATCATCAAACCAGGAGCCAAGACTCGAAAGGTTGAT
GACATCCGACAAAGGCTGCGAATGAGAGGCTTCCGATGCAACCTTGTCT

ACACACGTGCAGCATCAAGGTTGAATGTGATCCCATTATTTGCATCAAGG
AAACAAGCCTTAAGGTATCTTTCAGTTAAGTGGGGAATTGAGCTTTCCAA
AATGTTTGTCTTTGTTGGAGAAAGAGGAGATACGGATTATGAAGAACTAC
TGACTGGCCTTCATAAGACCCTTATCATAAGAGGCTCTGTGGAGTACGAA
AGGGAGAACTTTCTTCACAATGAAGATAGTTTTAAAAGAGAAGATATTGT
CCCACAAGAAAGCTCTAACCTACGCTTTGTGGAGGGAAATTATGAAGTTC
ATGACATCTCAGCAGCTTTAGAGACTCTATTGTTCAAATGA

Supplementary Data S2. Amino acid sequences of each *MeSPS* gene.

> ***MeSPS1***
MAGNDWINSYLEAILDVGPGLDDAKSSLLLRERGRFSPTRYFVEEVITGFDE
TDLHRSWVKAQATRSPQERNTRLENMCWRIWNLARQKKQLEGELAQRKA
KRHLEREKGRREATADMSEDLSEGEKGDAAGDVSVHGDSNRGRLPRINSV
DAMEAWANQQKGKKLYIVLISLHGLIRGENMELGRDSDTGGQVKYVVELAR
ALGSMPGVYRVDLLTRQVSAPDVDWSYGEPTEMLTLRNSEDFEDEMGESS
GAYIVRIPFGPKDKYIPKEHLWPHIPEFVDGALNHIIQMSKVLGEQIGGGKPIW
PVAIHGHYADAGDSAALLSGALNVPMLFTGHSLGRDKLEQLLKQGRLSRDEI
NSTYKIMRRIEAEEFSLDSSEIVITSTRQEIDEQWRLYDGFDPILERKLRARIK
RNVSCYGRSMPRMAIIPPGMEFHHIVPQEGDMDGELEGNEDHPTSPDPPIW
NEIMRFFTNPRKPMILALARPDPKKNITTLVKAFGECRHLRELANLTLIMGNR
DGIDEMSSTNASVLLSVLKLIDKHDLYGQVAYPKHHKQADVPDIYRLAAKTK
GVFINPAFIEPFGLTLIEAAAHGLPIVATKNGGPVDIHRVLDNGLLVDPHDQQ
SIADALLKLVADKQLWAKCRQNGLKNIHLFSWPEHCKTYLSRIASCKPRHPQ
WQKDNDGNDSSDTDSPGDSLRDLHDISLNLKFSLDGEKTGASGNDNSIESE
GDSADRKGKLENAVLAWSKGVLKNTQKTGYIDKGEQNNSSGKFPALRRRK
QIFVIAVDFDTISGLIEATKKIFDAVERERTEGSIGFILSTSLTMSEINSFLVTGG
FSPSDFDAFICNSGSELYYSNLNAEDNPFVVDFYYHSHIEYRWGGEGLRKTL
VRWVSSVIDKKAEKGEHIVMAAEQLSTNYCYAFKVQKPGMVPPVKELRKLL
RIQALRCHVIYCQDGTRINVIPVLASRSQALRYLYVRWGIELANVVVFVGESG
DTDYEGLLGGLHKSVILGGVCTSASSQLHANRNYPLADVIASDSPNIVRTTE
ECTSSDIRASLKQLACVKG

> ***MeSPS2***
MSEDLSEGERGDVHGEILSHGGSVRGRMPRISSVDVMENWAKQHKEKKLY
IVLISLHGLIRGENMELGRDSDTGGQVKYVVELARALGTTPGVYRVDLLTRQ
VSAPDVDWSYAEPTEMLNLINSENSMQEHGESSGAYIIRIPFGPKDKYIQKEL
LWPYIPEFVDGALNHIIQMSRVLGEQIGSGNPVWPVAIHGHYADAGDSAALL
SGALNVPMVFTGHSLGRDKLEQLLKQGRQSREEINATYKIMRRIEAEELTLD

ASEIIITSTRQEIEEQWRLYDGFDPVLERKLRARIKRGVSCHGRFMPRMIVIPP
GMEFHHIIPHDADMDGEDEEKFEDSPAAPDPPIWSEIMRFFSNPRKPMILAL
ARPDPKKNITTLVKAFGECRPLRELANLTLIMGNRDDIDEMSSTNASYLLSIIK
LIDKYDLYGHVAYPKHHKQSDVPNIYRLAAKTKGVFINPAFIEPFGLTLIEAAA
HGLPIVATKNGGPVDIHRVLDNGLLVDPHDQQSIADALLKLVSDKQLWARCR
QNGLKNIHLFSWPEHCKTYLARIASCRPRQPQWQRSEDGLRNEESDSPGD
SLRDIKDLSLKLSLDGDKNESGNLDNSLDTEENASDRKNVLGNNALTLSKDA
IRGAQNESIEKADNNICSSKFPSPRKRKYIFVIAVDGDTTSDSLEAIKIVVEVG
MKETSIGFILSTSMTISEVYSLLASGGLSPLDFDAFICNSGSEVYYPSSSNGD
TTGLPFVLDLDYHSHIEYHWGGEGLRRTLVRWAASINDKHGQEQIVVEDES
GSTAHCCAFKVKEPSMIPSFRELRKLMRIQGLRGHVIYCQNGSKINVIPVLAS
RSQALRYLYIRWGADLSKFVIFVGECGDTDYEGLLGGIHKSVILKGVGSNAR
KLHADRSYPLEDVTPFDSPNVVQSEGCNVNDIKQSLEKLGVLKV

> ***MeSPS3***
MAGNEWINGYLEAILDSGAGAIEEHKPAPPVNLSDRGHFNPTKYFVEEVVTG
VDETDLHRTWIKVVATRNTRERSSRLENMCWRIWHLTRKKKQLEWEGQQR
QASRRWEREEGRRDATEDLSEDLSEGEKGDIVGEMLLSETPRKKFQRNFS
NLEVWSEDKNEKKLYIVLISLHGLVRGENMELGRDSDTGGQVKYVVELARA
LARMPGVYRVDLFTRQVSSHEVDWSYGEPTEMLTSGSEDADGNEVGESS
GAYIIRIPFGPRDKYLRKELLWPYIQEFVDGALAHILNMSKVLGEQIGGDGPV
WPYVIHGHYADAGDSAALLSGALNVPMVLTGHSLGRNKLEQLLKQGRQSK
EDINSTYKIMRRIEAEELALDAAELVITSTKQEIEEQWGLYDGFDVKLEKVLRA
RSRRGVNCHGRYMPRMVVITPGMDFSNVVVQEDAPEVDGELASLIGGSDG
SPKAIPAIWSEVMRFLTNPHKPMILALSRPDPKKNITTLLKAFGECRPLRELA
NLTLIMGNRDDIDEMTGGNASVLMTVLKLIDKYDLYGLVAYPKHHKQYEVPDI
YRLAAKTKGVFINPALVEPFGLTLIEAAAHGLPMVATKNGGPVDINRALNNGL
LVDPHDQQAIADALLTLVSEKNLWHECRKNGWKNIHLFSWPEHCRTYLTRV
AACRMRHPQWQTDTPGDEMAAEESSLNDSLKDVQDMSLRLSIDGDKSSLN
ESLDYSAAAAGDPEIQAQVNQVMRKIKKPEAGPKDAEGGKNETGMSKYPM
LRRRRRLIVIALDCYGAEGAPENKMIQVVQYVIKAVRSDSLFARTSGIALATA
MPLSETVEFLASAKIQVNEFDALICSSGSELYYPGTYTEENGELLPDPDYAS
HIDYRWGCEGLKKTIWKLINTTEGAEQSKGSSSLIELDSKSSNAHCVAYWIK
DRKKVMKVHDLRQKLRMRGLRCHPMYCRSSTRMQIIPLLASRAQALRYLFV
RWRLNVANMYVILGETGDTDYEQMVAGAHKTIIMKGVVIKGSEELLRSMDLK
DDFVPKESRLIAHLSGESSASEIAEALKQVSKATGM

> ***MeSPS4***
MAGNDWINGYLEAILDVGSSLRKRNDGQLKISKFEDSKQKEDKSFSPTKYFV
EEVINSFDESDLHRTWVKVIATRNTRERSNRLENMCWRIWHLARMKKKIEW
DDAQRLARRRLEREQGRNDAADDLSELSEGEKEKGDTNLSEPVKNFSRINS

DMQIWSDEEKPRRLYIVLISMHGLVRGENMELGRDSDTGGQVKYVVELARA
LANTKGVYRVDLLTRQISSPEVDYSYGEPIEMLACPPDGSGSCGAYIVRIPC
GPREKYIPKESLWPYIPEFVDGALSHIVNMARALGEQVNGGKPTWPYVIHG
HYADAGEVASHLSGALNVPMVLTGHSLGRNKFEQLLKQGRLSKEDINVTYKI
MRRIEAEELGLDATEMVVTSTKQEIEEQWGLYDGFDIKLERKLRVRRRRGV
SCMGRHMPRMVVIPPGMDFSYVTTHDSLEGDLKSLIGPDRTQTKRNLPPIW
SEVMRFFTNPHKPTILALSRPDPKKNVTTLLKAFGECQRLRELANLTLILGNR
DDIEEMSNSSSVVLTTVLKLIDKYDLYGQVAYPKHHKQSEVPDIYRLAAKTKG
VFINPALVEPFGLTLIEAAAYGLPVVATKNGGPVDILKALNNGLLVDPHDQKAI
ADALLKLVADKNLWTECRKNGLKNIHRFSWPEHCRNYLSHIEHCRNRHPTS
RLEITPVPEEPMSDSLKDVEDLSLRFSIEGDPKLNGELDATTRQKKLIEAITQA
ASFNGNTNVTYSPGRRQMLFVIAVDCYDCNGKSTETFQEIIKNVMKAAGSCL
GLGRIGFVLSTGSSLQETMEALRYCPVNIEDFDAIICNSGSEMYCPWRDMVA
DLDYEAHVGYRWPGENVRSMAIRLAKVEDGAEDDVLEYVQASGSRSYSYII
KPGAKTRKVDEIRQRLRMRGIRCSLVYTRAASRLNVIPLFASRKQALRYLSV
RWGIDLSKIVVFVGEKGDTDYEELLAGLHKTLIMRGSVEYGSENLLCGQDGF
KREDIIPQESPSLRFVEENYENLSTALETLGIK

> ***MeSPS5***
MAANDWINGYLEAILDVGSSLRKRNEGKLNVVKFEDSKEKEDKSFSPTKYFV
EEVINSFDESDLHRTWVKVIATRNTRERSNRLENMCWRIWHLAREKKKIAW
DDAQRLARRQLELEQGRNDAADDLSELSEGEKEKGDANFSEPVKHFSRINS
DMQIWSDDEKPRHLYIVLISIHGLVRGENMELGRDSDTGGQVKYVVELARAL
ATTKGVYRVDLLTRQISSPDVDFSYGEPIEMLSCPPDGCGSCGAYIVRIPCG
PRDKYIPKESLWPYIPEFVDGALSHIMNMAKALGEQVNGGKPTWPYVIHGH
YADAGEVASHLSGALNVPMVLTGHSLGRNKFEQLLKQGRLSREDINATYKI
MRRIEAEELGLDASEMVVTSTKQEIEEQWGLYDGFDLQLERKLRVRRRRGV
SCMGRHMARMVVIPPGMDFSYVTTQDSLEGDLKSLIGSDRTQTKRNLPPIW
SEIMRFFTNPHKPTILALSRPDPKKNVTTLLKAFGECQRLRELANLTLILGNR
DDIEEMSNSSSVVLTAVLKLIDKYDLYGQVAYPKHHKQTEVPDIYRLAAKTKG
VFINPALVEPFGLTLIEAAAYGLPVVATKNGGPVDILKALNNGLLVDPHDQKAI
ADALLKLVADKNLWTECRKNGLKNIHRFSWPEHCRNYLSHIEHCRNRHPTS
RLEITSIPEEPMSDSLKDVEDLSLRFSIEGENKFNGELDAATRQKKLIEAISQA
TSSNGNASVTYSPGRRQMLFVIAVDCYDCNGKSTEAFQEITKNVMKAAGLC
VGLGKIGFILLTGSSLQETMEALRCCPVNIEDFDAIICNSGSEMYYPWRDMV
ADLDYEAHVDHRWPGENVRSVAIRLAKIEDGAEDDVLEYFQACGSRCYSYII
KPGAKTRKVDDIRQRLRMRGFRCNLVYTRAASRLNVIPLFASRKQALRYLSV
KWGIELSKMFVFVGERGDTDYEELLTGLHKTLIIRGSVEYERENFLHNEDSF
KREDIVPQESSNLRFVEGNYEVHDISAALETLLFK
